# Immune landscape of the affected brain in Rasmussen encephalitis

**DOI:** 10.1038/s41598-026-51295-3

**Published:** 2026-05-13

**Authors:** Giovanni Quinones-Valdez, Julia W. Chang, Shino D. Magaki, Harry V. Vinters, Noriko Salamon, Anthony C. Wang, Aria Fallah, Geoffrey C. Owens

**Affiliations:** 1 Department of Integrative Biology and Physiology, College of Life Sciences, University of California Los Angeles, Los Angeles, CA USA; 2 Division of Neuropathology, Department of Pathology and Laboratory Medicine, David Geffen School of Medicine, University of California Los Angeles, Los Angeles, CA USA; 3 Department of Neurology, David Geffen School of Medicine, University of California Los Angeles, Los Angeles, CA USA; 4 Department of Radiological Sciences, David Geffen School of Medicine, University of California Los Angeles, Los Angeles, CA USA; 5 Department of Neurosurgery, David Geffen School of Medicine, University of California Los Angeles, Los Angeles, CA USA

**Keywords:** Rasmussen encephalitis, T cells, Microglia, Single cell RNA sequencing, Endogenous retrovirus, Immunology, Neurology, Neuroscience

## Abstract

Rasmussen encephalitis (RE) is a rare neuroinflammatory disease characterized by intractable seizures and progressive brain atrophy that is usually confined to one cerebral hemisphere. Disease management involves anti-seizure medications and immunotherapy, although surgical resection remains the only effective treatment option to achieve seizure freedom. The presence of clonally expanded resident memory T cells in brain tissue removed to control seizures suggests the involvement of an autoimmune response in the etiology of the disease. Blocks of fresh brain tissue were obtained from three RE surgery cases (ages 5, 8, and 26 years at the time of surgery) and immune cells were isolated. Single cell RNA sequencing was used to define the types of immune cells present in the affected brain tissue and potential crosstalk between them, along with multiplex immunofluorescence immunostaining of sections from the same specimens. We matched T cell receptor sequences to T cell phenotypes and used ViralTrack software to search for evidence of activation of latent viruses in the immune cells. The immune cells isolated from the three RE cases comprised primarily activated microglia and resident memory CD8 T cells with fewer CD4 T cells, NK cells and monocyte-derived macrophages and dendritic cells. The majority of CD8 T cells expressed killer cell lectin-like receptors, and a virus responsive gene signature that included XCL1, TNFRSF9 and CRTAM, but also the exhaustion markers LAG-3 and TIM-3. Microglia expressed transcripts found in disease-associated microglia and transcripts associated with NLRP3 inflammasomes. We found no evidence for active latent viruses; however, we found endogenous HERV-K retrovirus sequences that were transcribed from multiple provirus insertion sites.

**Supplementary Information:**

The online version contains supplementary material available at 10.1038/s41598-026-51295-3.

## Introduction

Rasmussen encephalitis (RE) is a rare neuroinflammatory disease characterized by seizures and progressive brain atrophy that is usually confined to one cerebral hemisphere^1–3^, although bilateral cases have been described^4^. In a recent retrospective study of 160 RE cases, the median age of clinical onset was seven years (range 1 to 53 years) with the left cerebral hemisphere more often affected^5^. Disease management involves immunotherapy and anti-seizure medications (ASMs) although surgical resection remains the only effective treatment option to achieve seizure freedom^6,7^. The presence of clonally focused T cells in resected involved brain tissue suggests that RE may have an autoimmune component^8–11^, however, to date no T cell specific self-antigens have been identified. Comorbidity with several autoimmune diseases has indicated a potential heritable predisposition^12^, and, in support of this idea, we identified HLA class I and II alleles in a cohort of 24 RE surgical cases that have been linked to susceptibility to autoimmune diseases^9^. HLA class II nonsynonymous single nucleotide polymorphisms (SNP) were also found to be enriched in RE cases as well as several SNPs in other genes including those involved in an immune response^13,14^. Takahashi et al.^15^ identified SNPs in *CTLA4* and *PDCD1* that were overrepresented in a cohort of Japanese RE patients, which may negatively affect the function of these immune regulatory genes. Wang et al. identified a SNP in *IFITM3* in Chinese RE patients that may promote human cytomegalovirus (HHV5) persistence in the brain^16^. Epstein Barr virus (HHV4) has been proposed as a viral trigger for Multiple sclerosis (MS)^17^, and HHV4, HHV5, and HHV6 sequences have been reported in some RE brain specimens but not in others^18–23^. In support of a possible viral etiology, early treatment of two presumptive RE cases with ganciclovir was reported to stop seizures and resolve neurological deficits^21,24^. We found that many of the expanded T cell clonotypes in RE brain specimens were public suggesting that they may have been directed against a common infectious agent^9^. On the other hand, autoreactive T cell clonotypes may be present in healthy individuals^25^.

Co-existence of RE and focal cortical dysplasia (FCD) pathologies has also been documented in RE surgical cases suggesting that FCD could be involved in triggering the disease^26–28^. FCD is caused by somatic mutations in neural progenitors that occur during early brain development resulting in focal seizures^29^. It has been established that seizures can promote brain inflammation^30,31^, suggesting that seizure activity per se could trigger an inflammatory cascade leading to an autoimmune response.

From the recent large cohort retrospective study of RE cases^5^, it was found that the number of RE patients with twin siblings was higher compared with the general population, leading the authors to suggest that the risks of complications associated with preterm births, which are more prevalent in twin pregnancies^32^, may also be causative. For example, preterm birth is a risk factor for perinatal arterial ischemic stroke, which may in turn be linked to neuroinflammation and seizures^33,34^.

RNA sequencing of nuclei isolated from resected brain tissue confirmed earlier findings^35,36^ and revealed the heterogeneity of activated microglia in affected brain areas^37^. Based on the pathological stages of RE described by Pardo et al.^38^, activation of microglia was shown to occur prior to T cell infiltration^39^. In the present study we have sequenced RNA from individual immune cells isolated from three RE surgery cases and characterized the transcriptomes of both myeloid and lymphocyte populations including T cell receptors. We also present evidence for the reactivation of the endogenous retrovirus HERV-K in these cells.

## Methods and materials

This study was approved by the UCLA Institutional Review Board (IRB no. 18–001048). The patients or their parents or legal guardians provided informed consent for the use of the surgical remnant and blood for research purposes according to the Declaration of Helsinki. There were no exclusion criteria, and no reported comorbidities. All specimens were collected using the same standard operating procedures. De-identified patient information including age at seizure onset, age at surgery, and gender was collected with informed consent.

### Single cell RNA sequencing

Immune cells were isolated from blocks of fresh resected brain tissue as previously described^40^. In brief, brain tissue collected from the operating room in ice cold Hibernate® containing penicillin/streptomycin (120U/ml and 100ug/ml respectively, ThermoFisher, Carlsbad, CA) was transferred to magnesium calcium free Hanks Balanced Salt Solution containing HEPES (20 mM), glucose (5 mM) and penicillin/streptomycin (ThermoFisher), and finely minced on ice with spring scissors. Tissue fragments were transferred to RPM1 (ThermoFisher) containing 10% human serum (Phoenix Scientific, San Marcos, CA) and HEPES (20 mM) and digested overnight at room temperature with Type IV collagenase (~ 800U/ml) (Worthington Biochemical Corp. Lakewood, NJ) followed by fractionation on a 70%:30% Percoll® (Millipore Sigma, St. Louis MO) step gradient. The immune cells were collected from the interface between the two Percoll® steps, and two Chromium single cell gene expression libraries (5’ gene expression and TCR V(D)J) were prepared (10X Genomics, Pleasanton, CA) and sequenced on a NovaSeq 6000 instrument in a SP flow cell (2 × 50 bp) (Illumina Inc., San Diego, CA). Reads were demultiplexed and aligned to the Genome Reference Consortium Human Reference 38 (GRCh Build 38) and VDJ reference data (based on Ensembl 94 release) using Cell Ranger count and Cell Ranger VDJ pipelines (10X Genomics). The single cell RNA sequencing (scRNA-seq) data from the 5’ expression library were analyzed using the R package SingCellaR with the default settings^41^. The scRNA-seq data from each RE sample were combined using the integration function from Seurat^42^. Clusters of putative T cells and NK cells (n = 10,971) were then extracted, reintegrated using Harmony^43^, and reclustered. Clusters of putative myeloid cells (n = 12,877) were also extracted, reintegrated and reclustered. The R package Monocle 3.0^44^ was used to perform a trajectory analysis of the myeloid cells. The all_contig_annotations file from the Cell Ranger VDJ pipeline was used to assess the clonotype diversity in each RE sample, and to assign T cell phenotypes to the clonotypes based on the SingCellaR workflow. Spectratyping was performed with the immunarch R package^45^, and diversity estimates were calculated using the iNEXT R package^46^. Chord diagrams were made with the R package chorddiag^47^. Barcodes of the most abundant clonotype(s) in each sample were integrated into the SingCellaR object for visualization in UMAP projections. The Metascape web portal was used for pathway analysis^48^.

### Bioinformatics analysis of cell-cell communication

Myeloid cell and T cell/NK cell SingCellaR objects were converted to Scanpy (v1.11.5)-compatible.h5ad format. Datasets were subsequently merged, and raw counts were renormalized using log₂ counts per million (logCPM). Cell–cell communication (CCC) analysis was performed using LIANA + (v1.7.1)^49^. Multiple methods implemented within LIANA were applied, requiring that at least 20% of cells express the ligand or receptor gene and a minimum of 440 cells per interacting group (corresponding to 20% of the smallest cluster, monocytes-derived cells). The consensus ligand–receptor resource provided by LIANA was used, which integrates expert-curated databases including CellPhoneDB, CellChat, ICELLNET, connectomeDB2020, and CellTalkDB. LIANA’s built-in rank aggregation framework was applied alongside multiple individual CCC inference methods, including CellChat^50^, CellPhoneDB^51^, Connectome^52^, NATMI^53^ and SingleCellSignalR^54^ as well as summary metrics such as geometric mean and log₂ fold change (log₂FC)^55^. Interactions within the same cell type were excluded from downstream analyses. Method-specific filtering criteria were applied to identify high-confidence ligand–receptor pairs. For methods providing statistical significance (CellPhoneDB, geometric mean, and CellChat), p-values were adjusted using the Benjamini–Hochberg procedure, and interactions with adjusted p-values < 0.05 were retained. These interactions were ranked using method-specific magnitude metrics, including *lr_probs* (CellChat), *lr_means* (CellPhoneDB), and *lr_gmeans* (geometric mean). For other methods, the following thresholds were applied: log₂FC > 0.5; Connectome ligand or receptor z-score > 0; NATMI specificity weight > 0.1; and SingleCellSignalR *lrscore* > 0.5. For each method, interactions passing these thresholds were ranked according to their respective scoring metrics. The rank aggregation framework (*RankAggregate*) implemented in LIANA +, and based on the Robust Rank Aggregation (RRA) algorithm^56^, was used to derive the consensus specificity and magnitude rankings. This approach assigns a probabilistic score to each ligand–receptor pair based on its relative ranking across multiple methods, thereby prioritizing interactions that are consistently highly ranked. Finally, ligand–receptor pairs with a specificity rank ≤ 0.2 were retained, consistent with previous studies^57^.

### Identification of viral RNA using ViralTrack

For each sample, processing of the raw RNA sequencing reads was carried out using Umitools^58^. Initially, a whitelist of acceptable barcodes was generated, allowing for a Hamming distance of 1. Subsequently, the barcode sequences were extracted from the reads and incorporated into the read names. The next step involved mapping the reads to both the human genome (GRCh Build 38) and to a comprehensive viral genome database, encompassing over 14,000 distinct viral genomes. This mapping was performed utilizing the ViralTrack software package^59^. Only viral genomes meeting specific criteria were retained for further analysis. These criteria included a minimum coverage of at least 50 reads covering at least 10% of the entire viral genome sequence, and a sequence complexity of 1.3, as indicated by base composition entropy. Viral hits meeting these criteria were then assembled into contigs, which were considered to represent active regions of transcription within the viral genome. Subsequently, only those reads that mapped to contigs with a length of 200 bases or longer were retained. A custom Python script was employed to parse the alignments to both the human and viral genomes. Reads that mapped to both the human genome and a viral genome were assigned to the organism showing the highest alignment score. Finally, the read count of viral hits was quantified for each barcode, and barcodes were categorized as expressing the virus if they contained three or more reads mapped to a viral genome. The resulting viral expression data were integrated into the SingCellaR object for visualization in UMAP projections.

### Identification of HERV-K provirus insertion sites

We obtained the genomic coordinates of HERV-K provirus insertions from two previously published sources^60,61^. Coordinates from the Xue et al. study, originally reported in the hg19 (GRCh37) genome build, were converted to the hg38 (GRCh38) assembly using the liftover Python package (v1*.3.2*). Coordinates from both studies were then merged and manually curated to remove redundancies. To annotate the genomic context of each insertion, we used GENCODE v44 gene annotations. For each insertion, we recorded overlapping features including gene name, and region type (e.g., exon, intron, intergenic). Using a custom Python script, we quantified read coverage across the merged HERV-K insertion regions using the scRNA-seq data from the three RE patients, mapped to the reference hg38 (GRCh38) genome build. Reads were considered overlapping if they intersected a given region by at least 10 base pairs. We further filtered reads to retain only those also aligning to a reference HERV-K genome (NC_0022518) as determined from the prior ViralTrack analysis. Additional filtering criteria were applied to reduce mapping artifacts: retained reads were required to have an entropy score ≥ 1.0, an average Phred-scaled mapping quality ≥ 25, and a minimum aligned segment length of 20 bases. Chromosomal maps showing the sites of provirus insertion were made using the R package RIdeogram^62^.

### B cell receptor sequencing

Bulk genomic DNA was isolated from a frozen block of fresh brain tissue from RE patient 738, and from whole blood (Monarch® genomic DNA purification kit, New England Biolabs, Ipswich, MA). BCR sequences were obtained using the ImmunoSEQ® assay (Adaptive Biotechnologies, Seattle, WA), and clonal analysis was performed using the interactive web tool, ViCloD^63^.

### Immunocytochemistry

Paraffin embedded Sects. (5 um) were stained using Opal Fluorophore reagents (Akoya Biosciences, Marlborough, MA) and a Leica Bond RX auto-stainer (Leica Biosystems, Vista, CA) following antigen retrieval. In one panel, the following antibodies were used: CD20 (Opal 520), HLA-DR (Opal 570), CD4 (Opal 620) CD3 (Opal 690), and CD8 (Opal 780). In a second panel the following two antibodies were used, CD8 (Opal 570), and LAG-3 (Opal 690). Images were collected using a Leica Aperio Versa 200 Slide Scanning Microscope equipped with a 16-bit 5.5-megapixel fluorescence camera, and images were captured using Phenochart 2.0 (Akoya Biosciences), and colors were re-assigned. Images, as Tiff files, were imported into PHOTO-PAINT (Corel Corporation, Ottawa, Canada), to upscale the resolution to 600 dpi, and adjust tone curves. All figures were prepared in CorelDraw (Corel Corporation).

## Results

### Clinical and pathological description

**Fig. 1:**
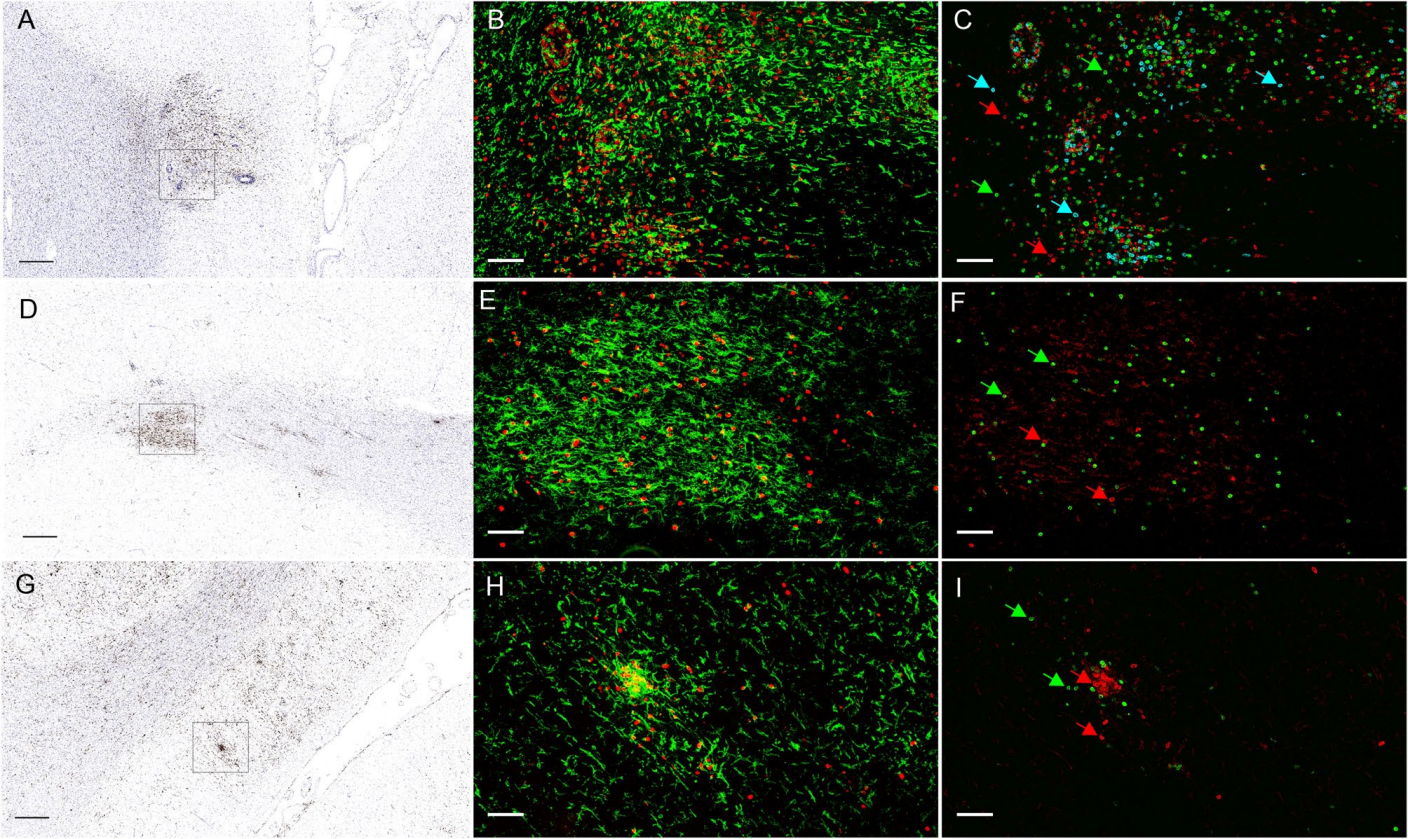
Immune cells in sections from the three surgical specimens (Patient 738, A, B, and C; patient 754, D, E and F; patient 769, G, H and I). Scans of multiplex immunostaining for HLA-DR, CD3, CD4, CD8, and CD20 were processed in PhenoImager HT 2.0. (**A**, **D**, and **G**) Low power images of HLA-DR expression rendered as a histochemical stain showing focal accumulation of activated myeloid cells (scale bar = 500 microns). (**B**, **E** and **H**) Higher power images of CD8 T cells (red), and HLA-DR positive myeloid cells (green). **C**, **F** and **I**) Images of the same area showing the CD8 T cells (green), CD4 T cells (red) and B cells (tourquoise). Colored arrows point to examples of the different immune cells (scale bar = 100 microns).

Three RE patients underwent surgery to control their pharmaco-resistant seizures (Table 1 and Fig. S1). In addition to anti-seizure medications (ASMs) two of the patients had received intravenous immunoglobulins (IVIG) in the months prior to surgery. Post-operatively all three patients were seizure free although still taking ASMs. We obtained formalin fixed paraffin sections, blood and fresh brain tissue from the surgical resections. A multiplex immunostaining protocol was used to visualize areas of inflammation in sections of resected brain tissue. Figure 1 shows areas of high HLA-DR expression marking microglia activation, and at higher magnification scattered CD8 and CD4 T cells amidst the activated microglia. In the selected section of brain from patient 738, B cells were intermingled with T cells, and in the case of patient 769 we found a dense cluster of CD4 T cells surrounded by microglia.

**Table 1:** Patient information.

Case ID	Gender	Age at seizure onset (yr)	Age at surgery (yr)	Seizure frequency	Affected hemisphere	Anti-seizure medication	Immuno- therapy
738	Male	21	26	~ 15 per day	Right	Gabapentin lamotrigine levetiracetam	IVIG 2 mo. before surgery
754	Male	5	8	~ 1 per day	Left	Brivaracetam clobazam clonazepam lacosamide	None
769	Female	2	5	~ 24 per day	Right	Lacosamide levetiracetam	IVIG 8 mo. before surgery

**Fig. 2:**
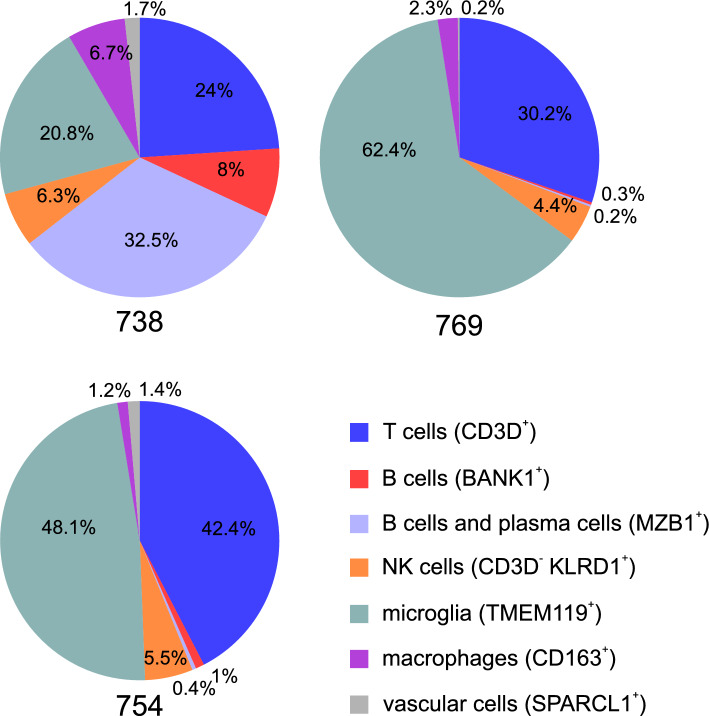
Pie charts depict the proportion of different cell types in the three RE surgery specimens after integration of the scRNA-seq data from each library.

Immune cells were isolated from each fresh brain specimen and single cell cDNA libraries were constructed targeting 10,000 cells. Both whole transcriptome and T cell receptor sequences were obtained, and the transcriptomic data from each patient were integrated using canonical correlation analysis (Fig. S2). The identity of the cells in each cluster was assigned based on known marker genes (Fig. S2), and from these assignments, the proportions of the different cell types from the three brain specimens were calculated (Fig. 2). Myeloid cells mainly comprised microglia and were clearly distinguishable from T cells and NK cells, except for a mixed cluster derived primarily from patient 769, which we attribute to cell doublets (Cluster 9, Fig. S2). Cluster 9 cells were excluded from further analysis.

### T cell phenotypes

**Fig. 3:**
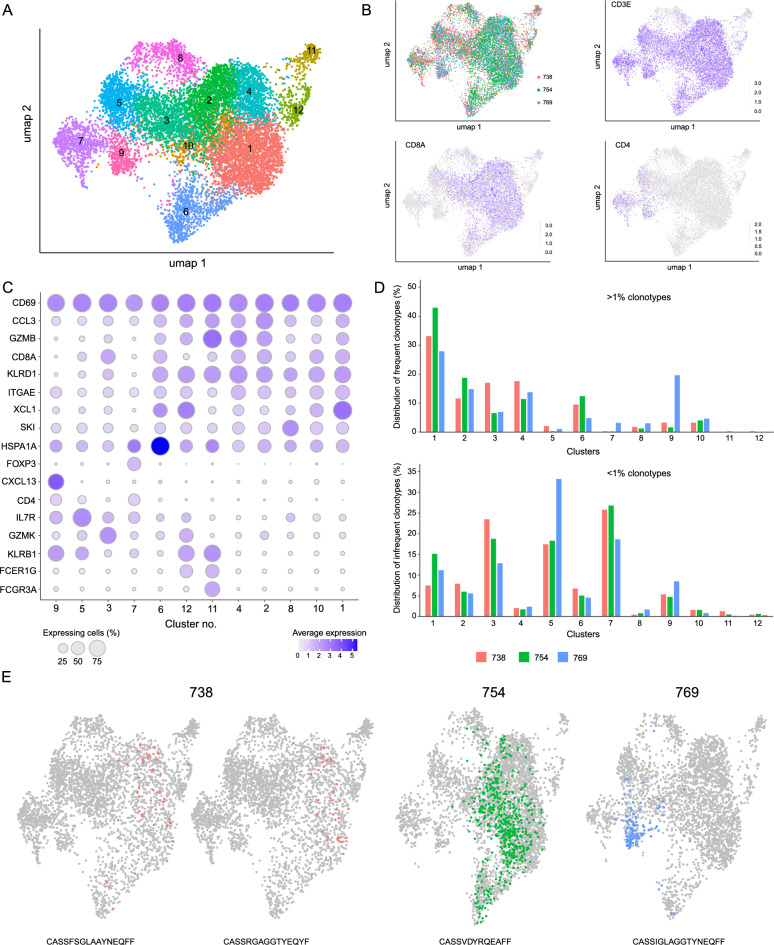
Phenotypes of the T cells and NK cells in the immune cell fractions that were isolated from the three RE surgery specimens. (**A**) UMAP of cell clusters based on the normalized counts of the most variable genes. (**B**) UMAPs showing the proportion of T cells and NK cells in the three surgical specimens and identification of the clusters comprising CD8 and CD4 T cells. (**C**) Bubble plot of selected genes that distinguish between the different T cell and NK cell clusters. (**D**) Frequency histograms showing the distribution of T cells clonotypes from the three surgical specimens among the T cell clusters. In the upper histogram the frequent clonotypes (> 1%) are plotted as a percentage of the total number of frequent clonotypes from each RE case. In the lower histogram the infrequent clonotypes (< 1%) are plotted in the same way. (**D**) UMAPS showing that the most frequent clonotypes in RE cases 738 and 754 are found in several CD8 clusters whereas the most frequent clonotype in RE case 769 is almost exclusively found in a single CD4 T cell cluster (see also Table S1).

The putative T cells and Natural Killer (NK) cells were extracted from the integrated dataset, and reclustered to further resolve cell phenotypes (Fig. 3A,B; Table S1). The majority of cells in all 12 clusters expressed CD69 a marker of resident memory T cells (T_RM_)^64^; a variable number of cells in each cluster also expressed ITGAE (CD103), a second T_RM_ marker^64^ (Fig. 3C). Based on differentially expressed genes, three of the 12 clusters contained CD4 T cells, of which cluster 5 was defined by the expression of *IL7R* and *KLRB1* mRNAs, cluster 7 by *FOXP3* mRNA expression and cluster 9 by *KLRB1* and *CXCL13* transcripts (Fig. 3C). In agreement with this interpretation GSEA using gene signatures for different T cell phenotypes^65^ indicated that cluster 5 contained CD4 memory T cells and cluster 7 comprised regulatory T cells (Tregs) (Table 2). Clusters 1–4, 6 and 10 comprised predominantly CD8 T cells, of which cluster 1 was further defined by high levels of XCL1 transcripts, cluster 3 by GMZK expression, and cluster 6 by high expression of heat shock proteins including HSP1A1 (Fig. 3C). GSEA with gene signatures for T cell phenotypes^65^, exhausted T cells^66^, and for virus responsive T cells^67^ indicated that cluster 1 CD8 T cells had a cytokine producing phenotype and were responsive to a virus whereas cluster 2 and 4 CD8 T cells were likely exhausted. Gamma delta T cells were found within the CD8 T cells clusters; presumably, their overall gene expression profiles were too similar to the alpha beta T cells (Fig. S3). Cluster 8 was defined by a higher level of SKI transcripts, a suppressor of CD103 expression in mice^68^, cluster 11 by FCGR3A (CD16) expression, and cluster 12 by XCL1 expression. GSEA with a published Natural killer (NK) cell gene signature^69^ indicated that clusters 11 and 12 contained NK cells (Table 2). In addition to the putative NK cells, the majority of CD8 T cells expressed *KLRD1* (CD94) mRNA, which encodes an NK cell C-type lectin that forms heterodimers with NKG2 molecules to either inhibit (NKG2A/B) or activate (NKG2C and NKG2E) NK and CD8 T cells by binding to HLA-E molecules^70^. Higher levels of transcripts encoding the activating receptors were found in the majority of NK cells and CD8 T cells compared with transcripts encoding the inhibitory receptor (Fig. S4). Similarly, *KLRK1* encoding NKG2D, another activating NK cell C-type lectin-like receptor, was also highly expressed whereas *KLRG1* encoding another inhibitory receptor was not (Fig. S4).

**Table 2:** GSEA analysis.

Cluster	Gene signature	Padj	NES
1	CD8 cytokine	0.0261	1.8833
	virus responsive	0.0041	1.8937
2	T_exhausted_	0.0021	1.8203
4	CD8 cytokine	0.0355	1.6735
	T_exhausted_	0.0340	1.5864
5	CD4 Naive/CM	0.0015	1.8320
	CD4/CD8	0.0034	1.7994
6	virus responsive	0.0490	1.8565
7	T_reg_	0.0017	1.6103
11	NK	0.0002	1.6981
	CD8 cytotoxic	0.0005	1.6877
12	NK	0.0148	1.8039

NES, normalized enrichment score.

Interrogating the dataset for expression of transcripts encoding co-stimulatory and co-inhibitory genes revealed that CD8 T cells expressed the co-stimulatory genes *ICOS*, *CRTAM* and *TNFRSF9*, but essentially no *CD27* or *CD28* (Fig. S5). In contrast to CD4 T cells, *LAG-3* was the dominant co-inhibitory gene expressed by CD8 T cells (Fig. S5); far fewer cells expressed the co-inhibitory gene *PDCD1*, which were largely confined to cluster 9 CD4 T cells. CD4 Tregs (cluster 7) expressed *CTLA4* and *TIGIT* mRNAs (Fig. S5), as well as the co-stimulatory genes *ICOS*, *TNFRSF9* (4-1BB), *CD27* and *CD28* (Fig. S5). Immunostaining with LAG-3 and CD8 antibodies confirmed the expression of LAG-3 by CD8 T cells (Fig. S6). The intense punctate staining in some CD8 T cells is consistent with the known intracellular storage of LAG-3^71^. We also examined the expression of selected transcription factors. More cells expressed *RUNX3*, *ZEB2*, *PRDM1* (Blimp), and *ZNF683* (Hobit) mRNAs than *TOX*, *TBX21* (Tbet) or *TCF7* mRNAs (Fig. S7). However, fewer cells assigned to the CD4 T cell clusters (5, 7 and 9) expressed *ZEB2* and *ZNF683* mRNAs compared with cells assigned to the CD8 T cell clusters. Very few cells in the NK cell clusters (11 and 12) expressed *BATF* mRNA (Fig. S7).

### Clonally focused CD8 T cells

**Fig. 4:**
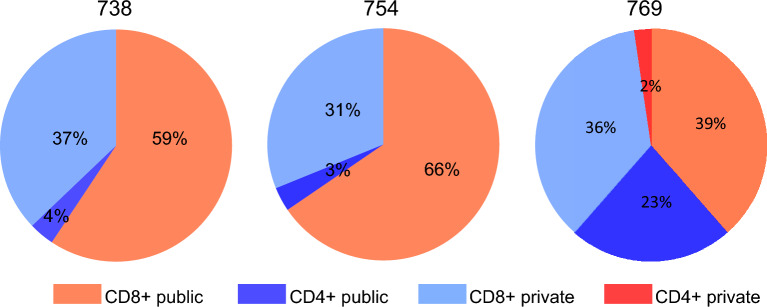
Pie charts depict the proportion of the frequent CD4 and CD8 T clonotypes (> 1%) that are either public or private (patient-specific).

We determined the number of different T cell clonotypes, defined by the Vbeta chain third complementarity region (CDR3) sequence, in the three RE brain specimens (Table S2). From Hill plots, *TRBV* and *TRBJ* gene usage, and CDR3 lengths it was clear that T cells in the brains of the three patients were clonally focused particularly in the brain samples from patients 754 and 769 (Fig. S8). Matching the barcodes from the TCR libraries to those comprising the T cell clusters showed that the most frequent clonotypes (defined as > 1%) were found in the CD8 T cell clusters with one exception (Fig. 3D). In patient 769 the most frequent clonotype could be ascribed to cluster 9 corresponding to a CD4 T cell subset defined by the expression of *KLRB1* and *CXCL13* mRNAs (Fig. 3E). In patients 738 and 754 the dominant clonotypes were CD8 T cells (Fig. 3E). We confirmed the phenotypes of 49 of the 54 most frequent clonotypes by extracting the CD3D^+^/CD3E^+^ CD4^+^ and CD8^+^ cells (≥ 1 normalized UMI) from the sparse matrix file of the reclustered T cell and NK cells and matching the barcodes to those of the top clonotypes (Table S2). To partition the top 1% into public and private clonotypes we compared them to Adaptive Biotechnologies’ immuneACCESS database (Table S2). As shown in Fig. 4 more than half of the abundant clonotypes were public. The Vbeta chain CDR3 amino acid sequence and *TRBV* and *TRBJ* genes of a rare clonotype in the blood of a single donor from large scale covid study^72^ were identical to the most abundant clonotype in patient 754 (Table S3). The CDR3 regions differed by three nucleotides indicating convergent selection^73^. Likewise, the most abundant clonotype in patient 769 was found in five individuals from an unpublished study of celiac disease available in the immuneACCESS database, although TRBV gene usage differed (Table S3).

We compared all the Vbeta CDR3 amino acid sequences to the VDJdb database^74^ and found that there were exact matches including *TRBV* and *TRBJ* gene usage to TCRs that recognize common viral epitopes. All these matching clonotypes were rare with one exception (Table S4). We implemented ClusTCR^75^ to identify clonotypes that may recognize the same epitopes. As shown in Table S5, several clonotypes were found that likely recognize the same antigen, notably HHV4 (IVTDFSVIK), and HHV5 (KLGGALQK).

### Expansion of B cell clones

**Fig. 5:**
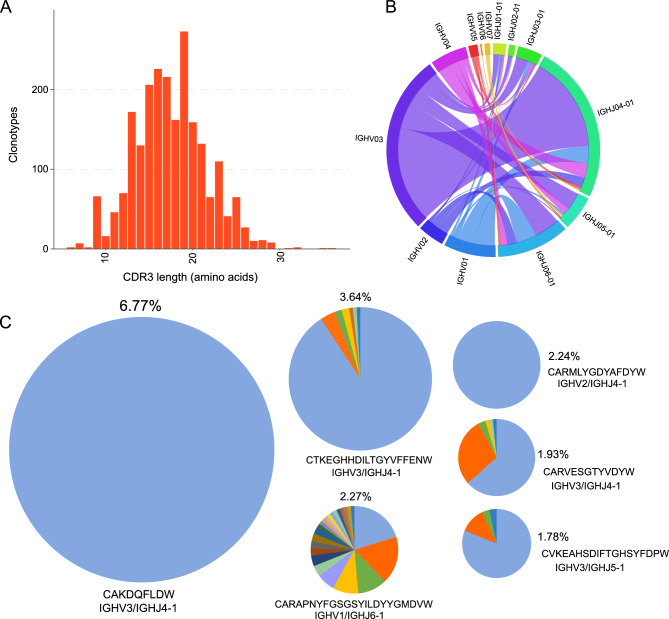
B cell clonotypes in RE case 738. (**A**) Frequency distribution of the CDR3 lengths in the sample of IgH sequences from sequencing genomic DNA isolated from part of the surgical specimen. (**B**) Chord plot showing IgH V and J gene usage. (**C**) Proportion of clonotypes that comprise the most frequent B cell clones (> 1%) resolved using ViCloD.

We found higher numbers of B cells and antibody-producing plasma cells in the brain specimen from Patient 738 (Figs.1, 2). Bulk immunoglobulin heavy chain (IGH) sequences were obtained from a fresh frozen piece of tissue from the same surgical specimen, and from whole blood collected at the time of the surgery. Repertoire analysis of IGH sequences identified the six most frequent clones (> 1%) in the brain and showed the extent of intra-clonal diversity (Fig. 5). The most abundant clone comprised a single IGH sequence that was not detected in the sample of IGH sequences from the blood suggesting local clonal expansion (Fig. S9).

### Myeloid cell phenotypes

**Fig. 6:**
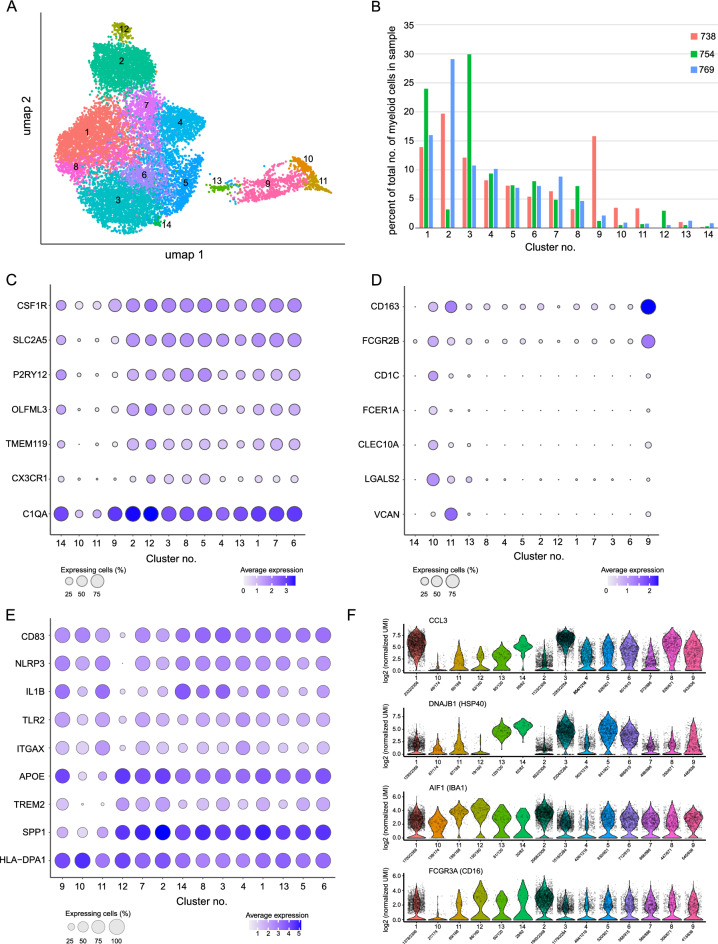
Phenotypes of the myeloid cells in the three RE surgical specimens. (**A**) UMAP showing the clusters of myeloid cells based on the expression of the most variable genes after integration of the scRNA-seq data. (**B**) Histogram showing the distribution of myeloid cell types among the three RE cases. (**C**) Bubble plot of selected genes that define microglia. (**D**) Bubble plot of genes that define monocyte-derived macrophages and dendritic cells. (**E**) Bubble plot of genes associated with activated microglia. (**F**) Violin plots of selected genes that distinguish between clusters of microglia.

Re-clustering the myeloid cells alone generated 14 clusters that were present in different proportions in each patient sample (Fig. 6A.B, Table S6). Pathway analysis associated clusters with different functions and protein complexes based on statistically significant differences in gene expression between clusters (Fig. S10). We interpreted 11 of the clusters as microglia based on the expression of the homeostatic glial marker genes *TMEM119*, *P2RY12*, *OFML3*, *SLC2A5*, and *CXC3CR1*^76–78^ (Fig. 6C). From the analysis of differentially expressed genes, cluster 9 appeared to define a population of macrophages as evidenced by high expression of *CD163* and *FCGR2B* (CD32) mRNAs by the majority of cells in the cluster^79,80^ (Fig. 6D). Cells in clusters 10 may comprise a dendritic cell population based on the expression of *CD1c*, *CLEC10A* and *LGALS2* (Galectin-2) mRNAs whereas cells in cluster 11 appeared to be a monocyte or monocyte-derived population based on *VCAN* (versican) gene expression^81^. The majority of the cells comprising clusters 9–11 were derived from patient 738 (Fig. 6B).

The microglia populations shared genes expressed by disease-associated microglia, specifically, *TREM2*, *APOE*, *CD83*, and *SPP1* (osteopontin)^76,82,83^, as well as genes directly associated with the NPLR3 inflammasome pathway (Fig. 6E)^35,84^. All myeloid clusters expressed high levels of HLA II molecules (Fig. 6E; see also Fig. 1). Cluster 3 microglia made up the largest fraction of myeloid cells in the brain specimen from Patient 754, whereas cluster 2 microglia were the most abundant myeloid cells in Patient 769 (Fig. 6B). From the differential gene expression analysis, it appeared that cluster 3 microglia expressed higher levels *DNAJB1* and *CCL3* transcripts than cluster 2 microglia, whereas cluster 2 microglia expressed higher levels of *AIF1* (IB1A), and *FCGR3A* (CD16) mRNAs (Fig. 6F). Less granular clustering of the myeloid cells and trajectory analysis using Monocle 3.0 indicated that microglia cells may transition from CD83^-^ cells expressing more IBA1 and CD16 to CD83^+^ cells expressing cytokines and heat shock proteins (Figure S11 and Table S7).

### Predicted myeloid cell: T cell communication

**Fig. 7:**
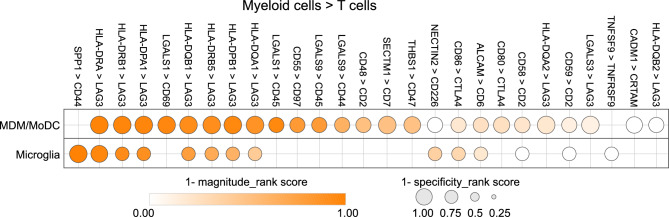
Predicted ligand: receptor interactions between myeloid cells and T cells. Bubble plot showing ligand: receptor pairings that were highly ranked by seven different methods implemented in LIANA +. Consensus rank specificity and magnitude scores are between 0 (highest rank) to 1 (lowest rank) (Table S8). The bubble plot depicts 1-rank specificity and 1-rank magnitude scores. MDM, monocyte-derived macrophages, MoDC, monocyte-derived dendritic cells.

We implemented LIANA + to investigate potential binding of ligands expressed by microglia and monocyte derived cells (Fig. 6D) to cognate receptors on T cells and NK cells. The results of this analysis are shown in Table S8 in which the same ligand: receptor pairings are highly ranked by seven different methods (see Methods and Materials for details). Microglia and monocyte-derived cells were predicted to interact with T cells (and NK cells) via ligand binding to several co-stimulatory and co-inhibitory receptors (Fig. 7). One of the strongest predictions as evidenced by the high specificity and magnitude rankings involved HLA class II proteins on myeloid cells and the co-inhibitory receptor LAG-3 on T cells/NK cells^85^. Galectin-3, encoded by *LGALS3*, and expressed by monocyte-derived cells was also predicted to bind LAG-3^85^. In addition, CD86 and CD80 binding to a second co-inhibitory receptor, CTLA, may also regulate T cell activity^86^. For microglia the SPP1: CD44 ligand: receptor pairing was ranked the highest. It has been reported that SPP1 (osteopontin) binding to CD44 suppresses T cell activation^87^.

The LIANA + analysis also highlighted potential activating interactions between myeloid cells and T cells and NK cells involving CD2, CD6, CD226, CRTAM, and TNFRSF9 (4-1BB)^88–92^. The CD226 ligand Netrin-2 also binds the co-inhibitory receptor TIGIT^93^, which we found was predominantly expressed by Tregs (Fig. S5). This interaction was not predicted probably because the number of TIGIT^+^ T cells did not meet the threshold that at least 20% of cells express the ligand or receptor gene. The expression of ligand genes in each myeloid cluster and the corresponding receptor genes in T cells and NK cells is shown in Fig. S12.

### Endogenous HERVK activation

**Fig. 8:**
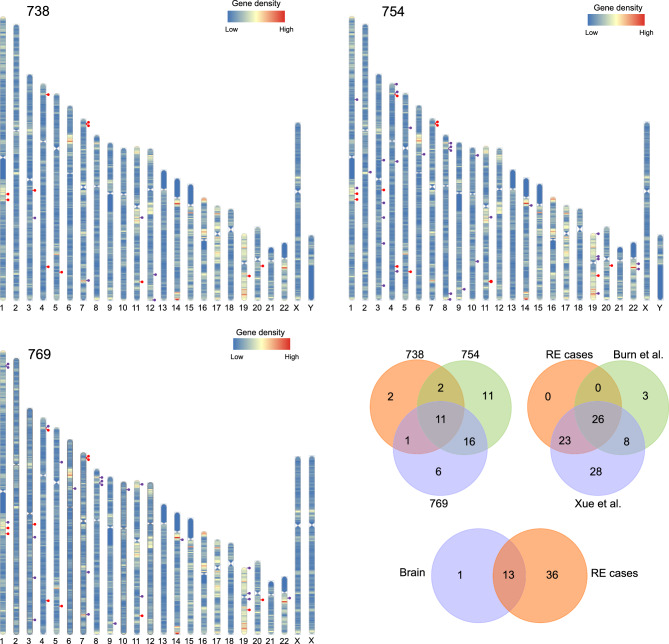
Locations of active HERV-K proviruses. (**A**) HERV-K transcripts from each brain specimen were mapped to known sites of HERV-K integration and displayed in an ideogram of the human karyotype showing gene density on each chromosome. Purple pins mark the patient-specific sites, and red pins mark the sites common to the three patients. (**B**) Venn diagrams showing the overlap between the three patients, the overlap with mapped sites of HERV-K integration from two published studies, and the overlap with active proviruses previously identified in normal brain cortex.

We used ViralTrack^59^ to search for viral transcripts in the scRNA-seq data from each RE brain specimen. Based on the filtering steps applied in the analysis (see Methods and Materials) we did not find evidence for any active exogenous viruses in the dataset. However, we did find evidence for activity of the endogenous retrovirus, HERV-K. The filtered HERV-K reads from each RE case could be uniquely mapped to previously identified sites of provirus insertion^60,61^ (Fig. 8; Table S9), indicating that multiple copies of HERV-K were transcriptionally active. Out of a total of 49 proviruses, 11 were in common between the three RE cases, and 13 were previously shown to be active in normal brain^60^. Associating reads with individual barcodes and imposing a threshold of at least three reads per barcode showed that HERV-K was active in more immune cells from patient 754 compared with the other two patients (Fig. S13). As shown in Fig. 8, the highest number of active proviruses was found in patient 754.

## Discussion

We have used scRNA-seq to characterize the immune landscape in brain areas affected by RE from three hemispherectomy surgery cases. The ages of the three patients, their treatment and timing of surgery were different, nevertheless it was possible to find the same types of immune cells in the resected brain tissue from each patient. Based on CD69 and CD103 antibody staining of T cells from other RE cases we previously reported that the majority of T cells in resected brain tissue were T_RMs_ indicating that they had entered the brain at an earlier time point before the surgery in response to an immune challenge, and then had remained there^94^. In the present study we found that essentially all CD4 and CD8 T cells and NK cells expressed *CD69* and variable levels of *ITGAE* (CD103) mRNA. We also documented the expression *RUNX3*, *PRDM1* (Blimp-1), and *ZNF683* (Hobit), transcription factors that have been implicated in establishing T_RMs_^95,96^.

The bioinformatics pipeline that we employed identified distinct populations of CD4 and CD8 T cells. The major CD8 T cell populations, which accounted for most of the high frequency clonotypes, comprised cells that expressed genes associated with an effector phenotype, as evidenced by the expression of the granzyme gene *GZMB*, and the NK cell activating C-type lectin-like genes *KRLD2*, *KLRKC2*, *KLRC3* and *KLRK1*. We also detected a population of CD8 T cells characterized by high levels of *GZMK* transcripts. Unlike Granzyme B, Granzyme K can induce non-caspase dependent cell death by cleaving both cytotoxic and non-cytotoxic intracellular proteins^97^. GZMK^+^ CD8 T cells have been found in tissues and lesions associated with several different autoimmune diseases and are assumed to be autoreactive^98,99^.

Exhausted T cells have been extensively described in chronic infections and cancers^100^ and are characterized by high expression of co-inhibitory molecules including PD1, LAG-3, TIM-3, and TIGIT^101^. The persistence of disease-triggering self-antigens in autoimmune diseases is also expected to generate an exhausted or dysfunctional state. GSEA indicated that there were exhausted T cells among the CD8 T cells found in affected RE brain tissue; notably LAG-3 transcripts were detected in about half the CD8 cells. We confirmed that LAG-3^+^ CD8 T cells were present in a section of resected brain tissue, consonant with a recent report^102^. In this study it was found that the number of T cells expressing PD-1 was significantly higher in tissue sections from later clinical stages compared with early stages of the disease, potentially reflecting an increase in the proportion of exhausted T cells. This difference between clinical stages could explain why we found few T cells expressing *PD-1* transcripts in our dataset. In a mouse model of type 1 diabetes, it was shown that LAG-3^+^ intra-islet CD8 T cells had an exhausted-like phenotype^103^. Deleting *Lag3* accelerated the development of diabetes, strongly implicating LAG-3^+^ CD8 T cells in the disease^104^. LAG-3 binds HLA class II molecules, and also binds to other ligands including the product of the *LGALS3* gene, Galectin 3^85^. We found HLA class II transcripts in essentially all myeloid cells, and LIANA + predicted a specific interaction with LAG-3^+^ T cells, and also between LAG-3 and Galectin-3 on monocyte-derived cell, which suggests that the effector function of CD8 T cells might be held in check by these interactions. A LAG-3 agonist antibody has been developed as a potential treatment for autoimmune diseases^105^. An immunosuppressive interaction between microglia and T cells and NK cells may also involve osteopontin binding to CD44^87^. *SSP1* and *CD44* genes were expressed in the majority of microglia and T cells and NK cells respectively.

There are a number of reports documenting the presence of active HHV4 and HHV5 in brain areas affected in RE^20,22,23^. We found exact HLA-matched TRB sequences in the three RE brain specimens that have been shown to recognize immunodominant peptides from HHV4 and HHV5 indicating that the three patients may have been exposed to these viruses leading to a subclinical immune response and viral latency in circulating monocytes and B cells^106–108^. Although the HHV4 and HHV5-specific T cell clonotypes did not correspond to the most abundant clonotypes in each surgery case, we considered the possibility that a reactivated herpes virus was present in peripheral immune cells present in the brain^109^. We used ViralTrack to search for viral transcripts in our dataset but did not find any herpes virus transcripts. We cannot exclude the possibility of an active virus in other brain cells or that infected cells had already been eliminated leaving behind resident memory T cells^110^.

Over half of the frequent clonotypes (> 1%) were public suggesting that some of the expanded T cells in the brain might result from exposure to a common infectious agent, and raising the possibility of molecular mimicry if these T cells also recognize self-antigens^111^. In patient 769 the Vbeta CDR3 amino acid sequence of the most abundant clonotype only matched a rare clonotype in the blood of seven individuals diagnosed with celiac disease, and in patient 754 the Vbeta CDR3 sequence and TRB and TRJ genes exactly matched a rare clonotype in the blood of a single uninfected individual from a recent SARS-CoV-2 study (Table S3)^72^. Given the limited sharing of these two clonotypes with other individuals it is possible that they may have escaped peripheral tolerance mechanisms and be directly autoreactive.

In our search for viral transcripts, we found HERV-K sequences in our scRNA-seq data indicating that this endogenous retrovirus was activated in immune cells from each RE patient. Mapping the virus contigs in individual cells showed that more microglia expressed HERV-K in patient 754 than in the other two patients. It has been reported that the presence of HERV-K transcripts in glioblastomas, teratoid rhabdoid tumors and in spinal cord neurons of ALS patients has potential disease-modifying effects^112–114^. Among the eleven proviruses active in all three patients, the provirus at Chr7p22.1a, ERK-6 (HERV-K108) contains full length open reading frames for the gagpol and envelope (env) genes; a mutation in the reverse transcriptase domain renders the virus defective^115^. The envelope gene from this provirus has been shown to be translated and expressed on the cell surface of transfected cells^116^. Whether the env protein is expressed in affected brain areas in RE remains to be determined. It has been reported that a GU-rich sequence in the *env* gene RNA can trigger TLR8-dependent death of neurons indicating that any pathological consequence of HERV-K activation might not depend on a translated open reading frame^117^. We should note that HERV-K sequences, including 13 we identified in the RE specimens, have also been found in transcript data from normal brain samples in the GTEx database including ERK-6^60,118^.

CD4 T cells could be assigned to three different phenotypes Tregs, IL7R^+^ memory cells, and a population of T cells distinguished by the expression of *CXCL13* and *KLRB1* (CD161) transcripts. CXCL13 is a chemoattractant for B cells, and CD4 cells producing this cytokine have been associated with the formation of tertiary lymphoid structures (TLS)^119^. In Fig. 1, a cluster of CD4 + T cells surrounded by HLA-DR + myeloid cells could correspond to this CD4 T cell subtype and define a developing TLS. Such ectopic structures have been found in affected tissues in autoimmune diseases^120^, and CD4 T cells producing CXCL13 are present in synovial fluid in Rheumatoid arthritis patients^121^. In Patient 769 the most frequent clonotype was in fact this CD4 T cell subtype (Table S2). On the other hand, we found the highest number of B cells and plasma cells in Patient 738. A higher number of monocyte-derived cells was also present in the sample of brain tissue from this patient. Sequencing immunoglobulin heavy chains revealed that these cells comprised expanded clones implying a specific humoral response to an antigen(s) in the brain of this patient. Patient 738 was the oldest surgery case at 26 years old, and seizure onset was documented five years earlier according to his clinical history (Table 1). However, the patient also experienced seizures when he was four years old and was treated with ASMs for two years. We speculate that the onset of seizures later in life may reflect a reactivation of resident T cells and a de novo humoral response. Autoantibodies directed against synaptic proteins have been found in the blood from some RE patients^122–124^, which has led to the use of an anti-CD20 antibody to try to control the disease^125–128^.

TIGIT expression by Tregs suggested that they were likely to be highly suppressive effector Tregs^129^. Therefore, their presence in the affected areas of the brain may play a pivotal role in suppressing the activity of the clonally focused CD8 T cells found in the same brain areas. It has been proposed that Treg dysfunction contributes to the etiology of autoimmune diseases^130,131^, and ways to increase Treg numbers and efficacy in autoimmune diseases are being actively pursued^130,132,133^. In addition to TIGIT, our analysis indicated that Tregs were regulated by several other co-stimulatory and co-inhibitory proteins including ICOS, CD27, CD28, and CTLA4 (Fig. S6). CTLA4 is constitutively expressed by Tregs and competes with CD28 for binding to CD86 and CD80 on antigen presenting cells to suppress conventional T cells by cell intrinsic and extrinsic mechanisms^134,135^, that may also involve engagement of the TCR^136^. We detected CD86 transcripts in the majority of myeloid cells and CD80 transcripts in monocyte-derived cells. LIANA + predicted binding to CTLA4 suggesting that myeloid cells may regulate Treg cells present in the affected brain area via CTLA4 binding to CD80 and CD86^86^.

Most of myeloid cells that we isolated from affected brain tissue were microglia and were distinguished from smaller populations of macrophages and monocyte-derived immune cells (monocyte-derived macrophages and monocyte-derived dendritic cells). Based on the default resolution parameter used, ten clusters of microglia were generated by the Louvain algorithm, although all cells expressed known homeostatic microglia marker genes, and genes associated with activated microglia. Trajectory analysis indicated that microglia transitioned from cells expressing higher transcript levels of genes associated with phagocytosis, *FCGR3A* (CD16) and *AIF1* (Ib1a), to cells expressing higher levels of proinflammatory cytokines, CCL3/4 and CD83 and to cells expressing CD83, cytokine and heat shock protein transcripts, although the latter might be attributable to stress induced during ex vivo dissociation of the brain tissue^137^. On the other hand, a subpopulation of microglia characterized by CD83 and heat shock protein gene expression was found to be selectively depleted in the substantia nigra of Parkinson disease patients implying a protective function that may also pertain to RE^138^.

Most microglia expressed HLA class II, *ITAGX* (CD11c), and *CD86* transcripts indicating competency as antigen presenting cells^139,140^. As previously discussed, microglia could regulate CD8 T cell activity via LAG-3 binding to HLA class II molecules. In a mouse model of type 1 diabetes, a *Lag3* mutation that disrupts binding to stable MHC II causes the same disease exacerbation as a *Lag3* null mutation^141^.

## Conclusion

Our analysis highlights the complexity of the immune landscape in brain areas affected by RE and supports the involvement of clonally expanded antigen experienced resident memory CD8 T cells in the etiology of RE. The presence of resident CD4 Tregs in the affected brain area suggests that they may be playing a role in restraining the activity of the conventional T cells. Likewise, microglia and monocyte-derived cells may regulate T cell activity via co-inhibitory and co-stimulatory receptor binding. However, further work involving expanded multiplex antibody panels and spatial analysis will be necessary to validate predictions of ligand: receptor binding between myeloid cells and T cells based on the mRNA expression data. Activation of HERV-K proviruses in the affected brain area may be unrelated to the etiology of RE although it is conceivable that translation of viral proteins from this endogenous virus could generate neo-antigens to which the patient’s immune system may react.

## Supplementary Information

Supplementary Information 1.

Supplementary Information 2.

Supplementary Information 3.

Supplementary Information 4.

Supplementary Information 5.

Supplementary Information 6.

Supplementary Information 7.

Supplementary Information 8.

Supplementary Information 9.

Supplementary Information 10.

Supplementary Information 11.

Supplementary Information 12.

Supplementary Information 13.

Supplementary Information 14.

Supplementary Information 15.

Supplementary Information 16.

Supplementary Information 17.

Supplementary Information 18.

Supplementary Information 19.

Supplementary Information 20.

Supplementary Information 21.

## Data Availability

NCBI Gene Expression Omnibus accession number GSE312319.

## Abbreviations

ASM: Anti-seizure medication
BCR: B cell receptor
CDR3: Complementarity determining region 3
FCD: Focal cortical dysplasia
HLA: Human leukocyte antigen
HHV: Human herpes virus
IgH: Immunoglobulin heavy chain
LIANA: Ligand receptor analysis framework
MS: Multiple sclerosis
RE: Rasmussen encephalitis
scRNA-seq: Single cell RNA sequencing
TCR: T cell receptor
UMI: Unique molecular identifiers
UMAP: Uniform manifold approximation and projection
