# Supplementary material for: Immune landscape of the affected brain in Rasmussen encephalitis
Source: Sci Rep. 2026 May 13;16:21957. doi: 10.1038/s41598-026-51295-3 (PMC13365386; doi:10.1038/s41598-026-51295-3)
Supplement: Supplementary file 1 — Supplementary Information 1. [file 41598_2026_51295_MOESM1_ESM.pdf]

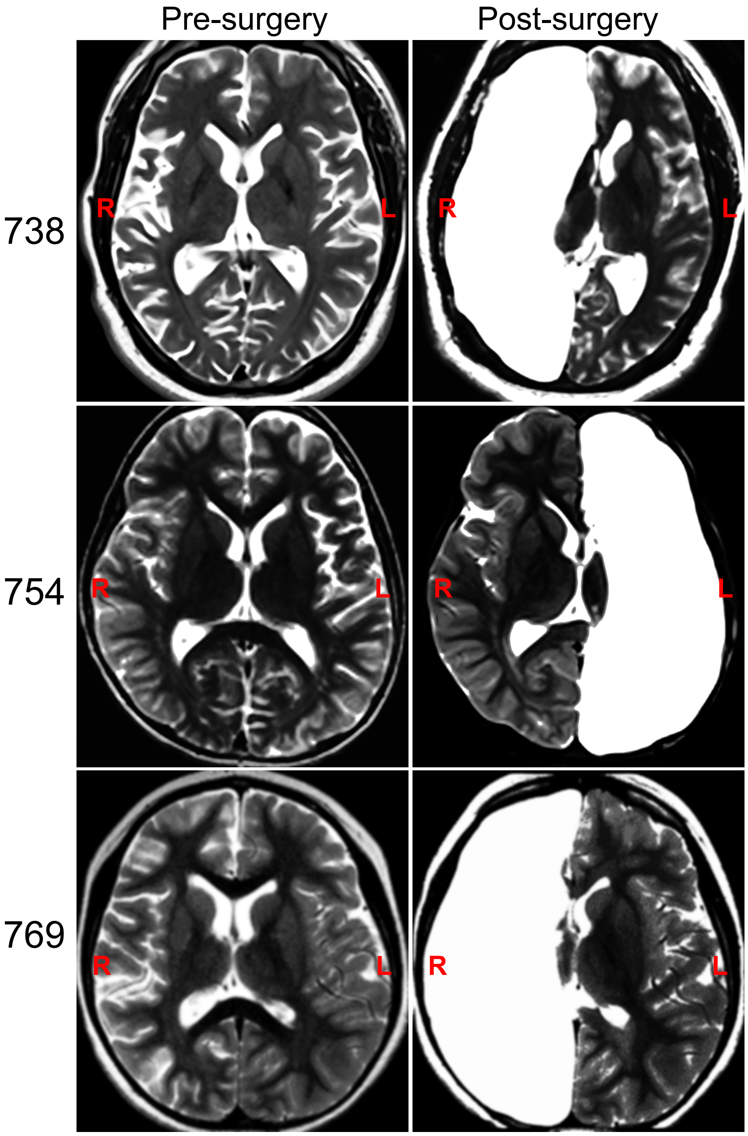


**Fig. S1:** T2 MRI scans of the three RE surgery cases, 738, 754, and 769 before and after surgery showing the affected cortical area that was removed.
