# Supplementary material for: Immune landscape of the affected brain in Rasmussen encephalitis
Source: Sci Rep. 2026 May 13;16:21957. doi: 10.1038/s41598-026-51295-3 (PMC13365386; doi:10.1038/s41598-026-51295-3)
Supplement: Supplementary file 2 — Supplementary Information 2. [file 41598_2026_51295_MOESM2_ESM.pdf]

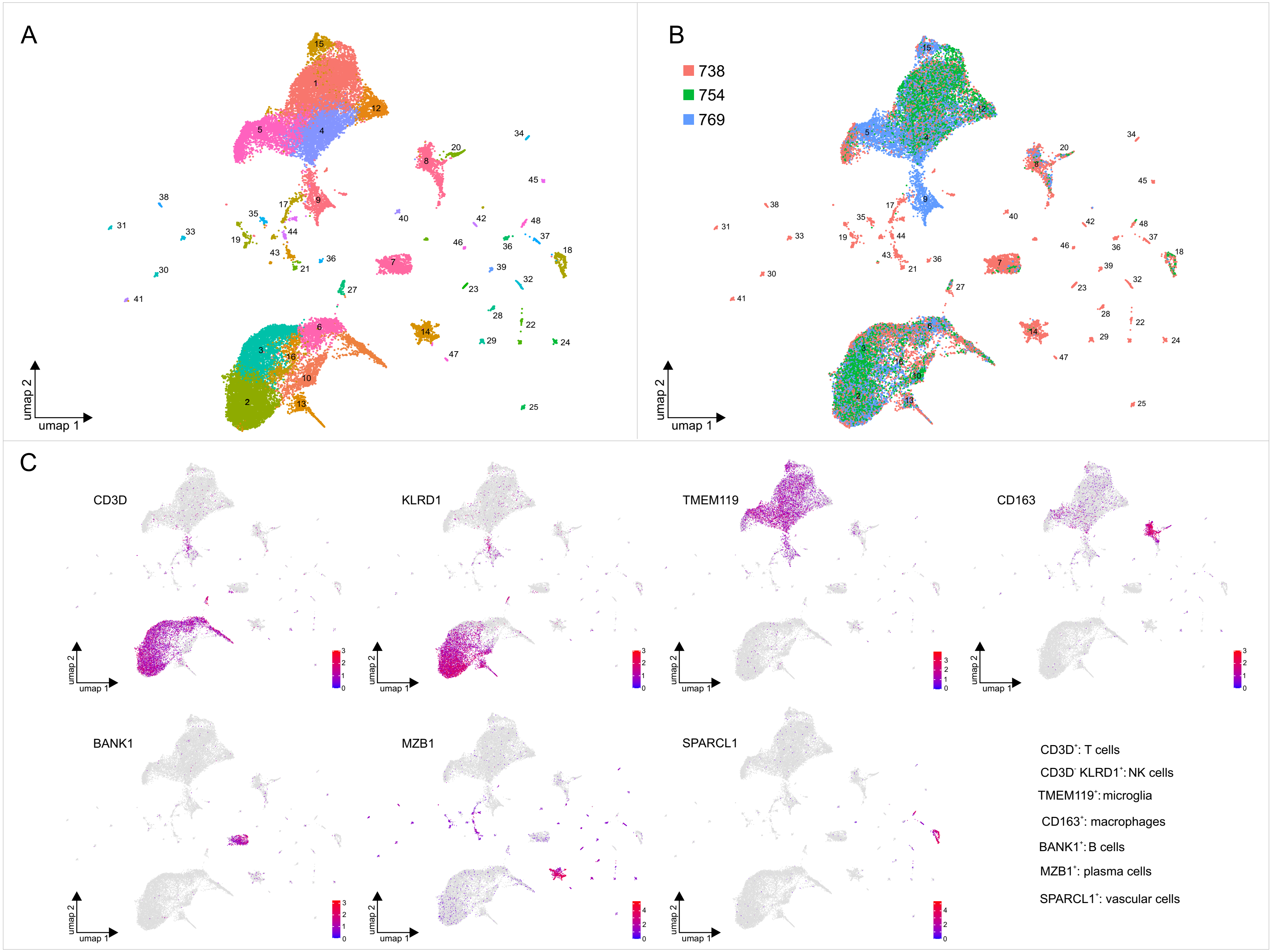

Fig. S2: Cell clusters obtained by integrating the scRNA-seq gene expression data from the three surgical specimens. (A) UMAP showing the number of clusters. (B) UMAP showing the distribution of the cells from each surgical specimen among the clusters. C) Assignment of cell types to each cluster based on the expression of a specific marker gene.
