# Supplementary material for: Immune landscape of the affected brain in Rasmussen encephalitis
Source: Sci Rep. 2026 May 13;16:21957. doi: 10.1038/s41598-026-51295-3 (PMC13365386; doi:10.1038/s41598-026-51295-3)
Supplement: Supplementary file 3 — Supplementary Information 3. [file 41598_2026_51295_MOESM3_ESM.pdf]

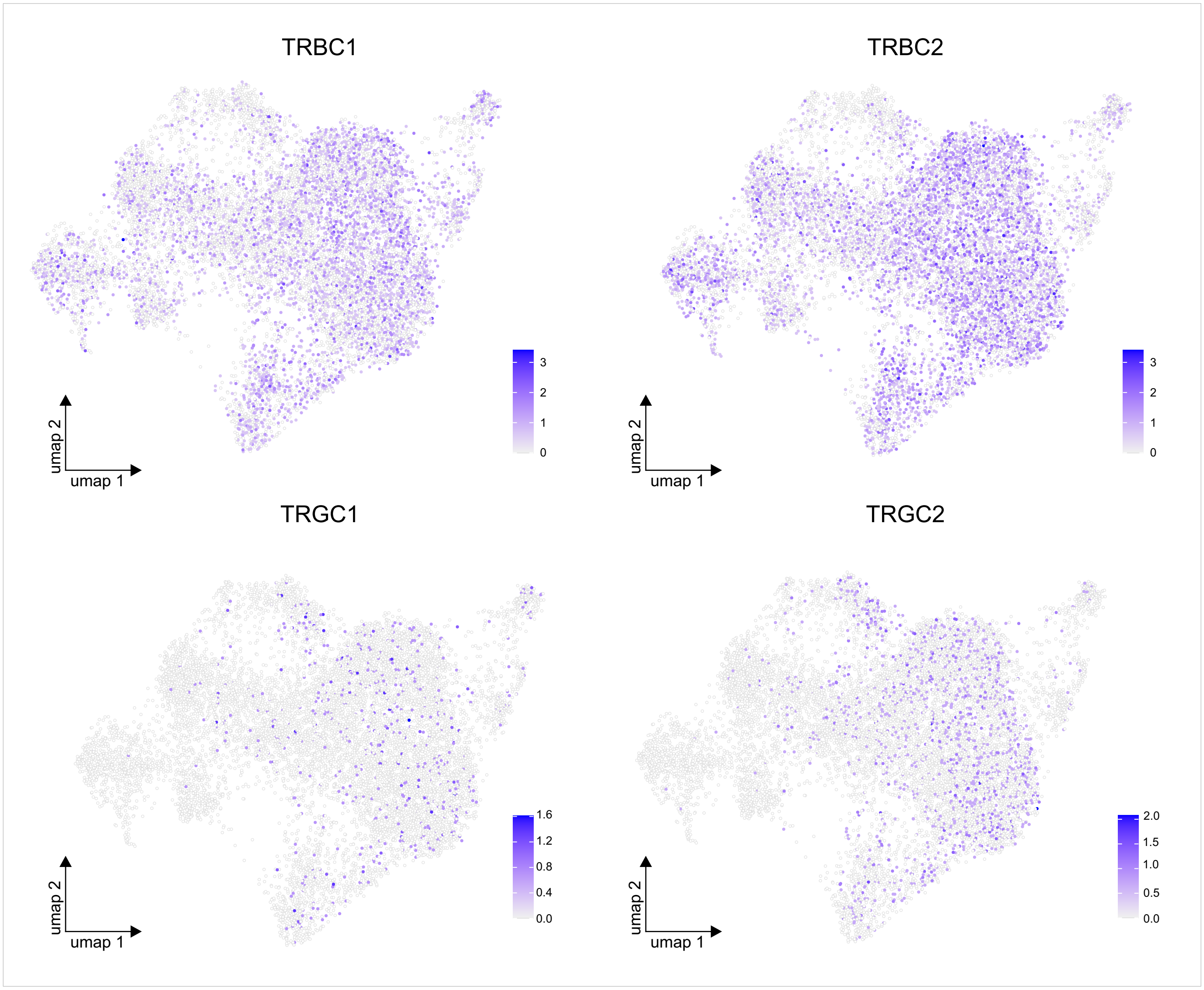

Fig. S3: Feature plots showing the normalized expression of TCR constant region genes in a UMAP of the T cells and NK cells extracted from the integrated scRNA-seq gene expression data from the three surgical cases.
