# Supplementary material for: Immune landscape of the affected brain in Rasmussen encephalitis
Source: Sci Rep. 2026 May 13;16:21957. doi: 10.1038/s41598-026-51295-3 (PMC13365386; doi:10.1038/s41598-026-51295-3)
Supplement: Supplementary file 4 — Supplementary Information 4. [file 41598_2026_51295_MOESM4_ESM.pdf]

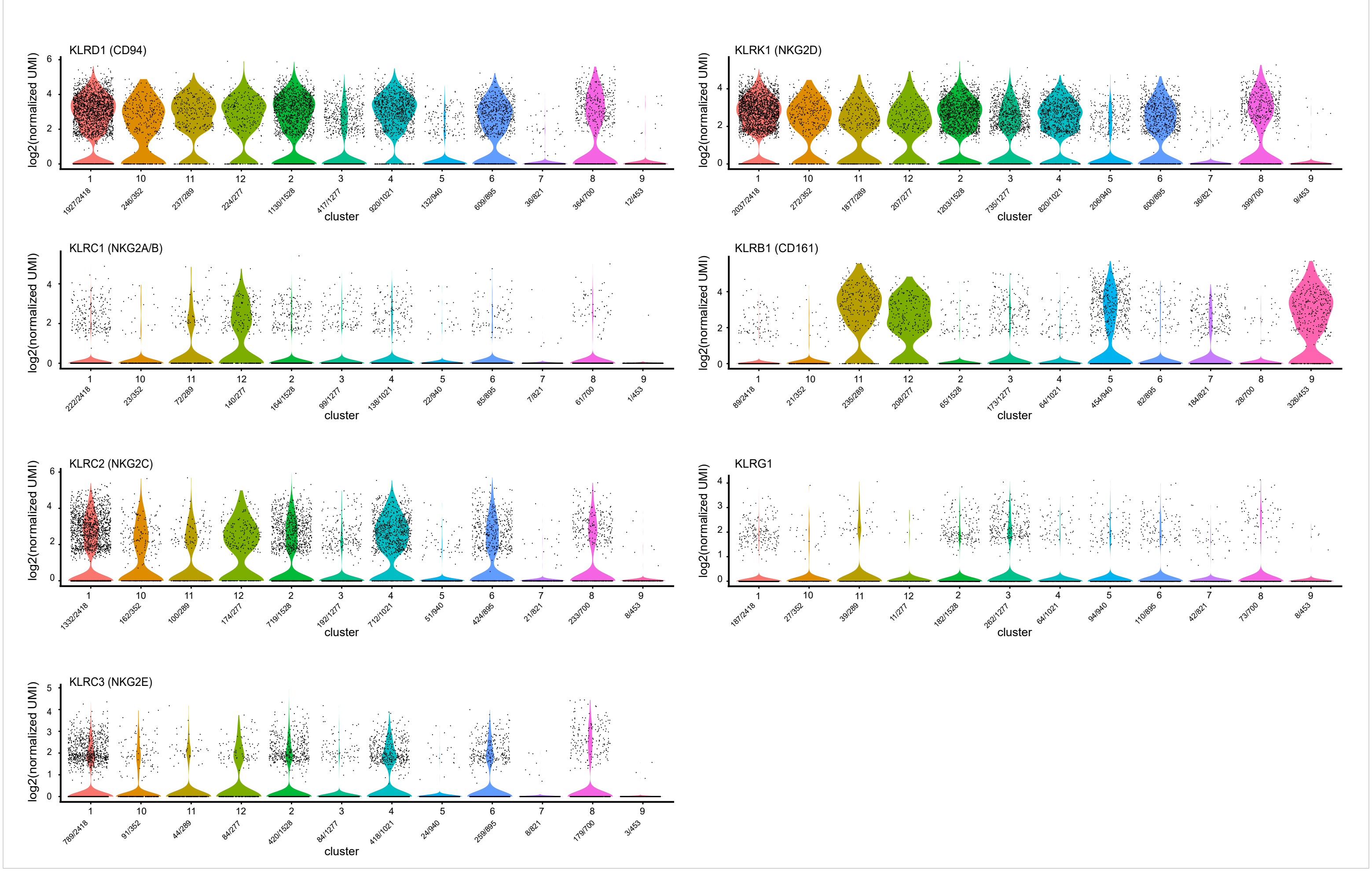

Fig. S4: Violin plots showing the normalized expression of genes encoding killer cell lectin-like receptors (KLR) in the clusters of T cells and NK cells. KLRD1 (CD94) and KLRK1 transcripts are predominantly found in CD8 T cells along with KLRC2 (NKG2C), the activating binding partner of KLRD1. In contrast there is lower expression of inhibitory receptors except KLRB1 which is expressed predominantly in NK and CD4 T cell clusters.
