# Supplementary material for: Immune landscape of the affected brain in Rasmussen encephalitis
Source: Sci Rep. 2026 May 13;16:21957. doi: 10.1038/s41598-026-51295-3 (PMC13365386; doi:10.1038/s41598-026-51295-3)
Supplement: Supplementary file 5 — Supplementary Information 5. [file 41598_2026_51295_MOESM5_ESM.pdf]

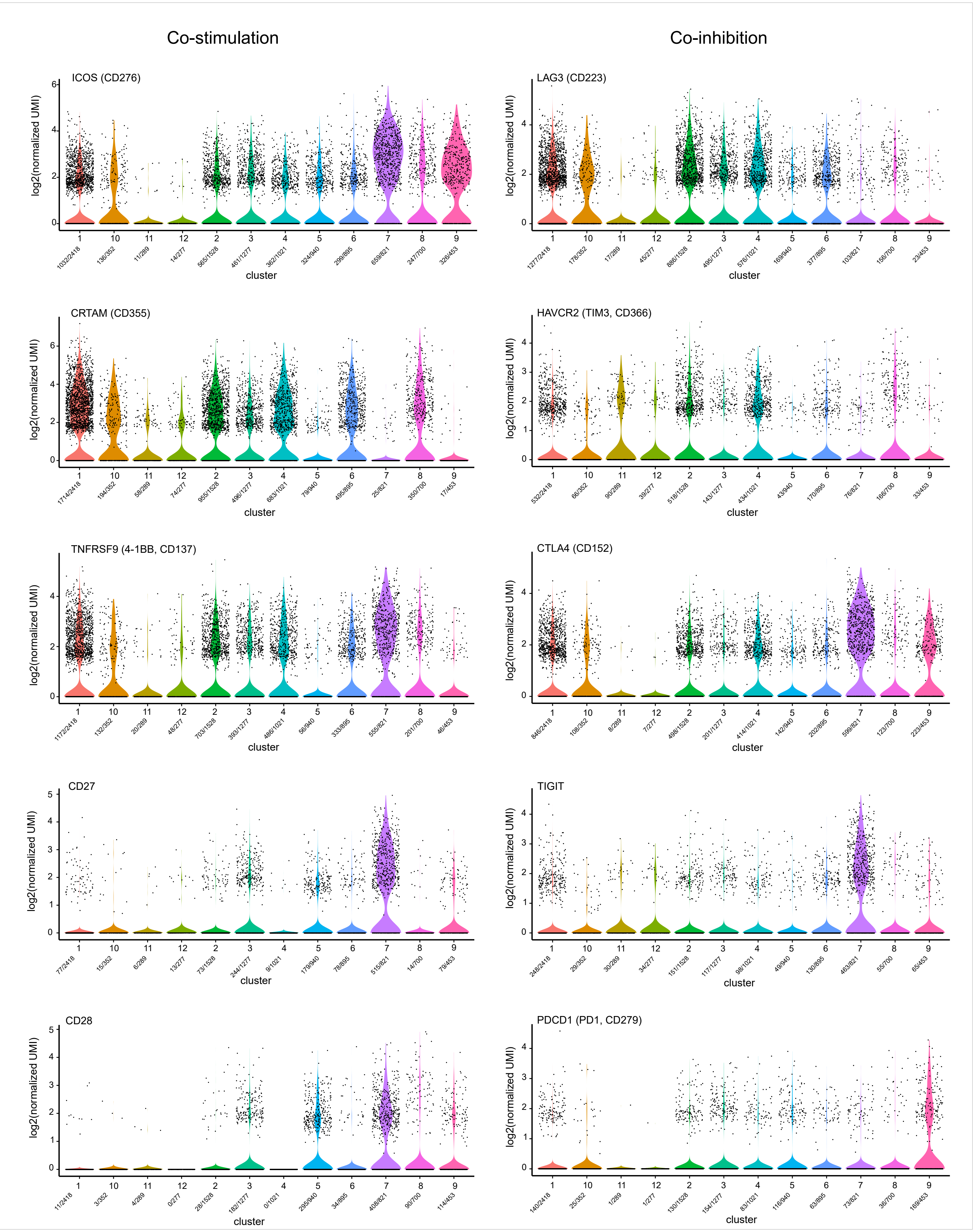


**Fig. S5:** Violin plots showing the normalized expression of genes encoding selected co-stimulatory and co-inhibitory receptors in the clusters of T cells and NK cells.
