# Supplementary material for: Immune landscape of the affected brain in Rasmussen encephalitis
Source: Sci Rep. 2026 May 13;16:21957. doi: 10.1038/s41598-026-51295-3 (PMC13365386; doi:10.1038/s41598-026-51295-3)
Supplement: Supplementary file 6 — Supplementary Information 6. [file 41598_2026_51295_MOESM6_ESM.pdf]

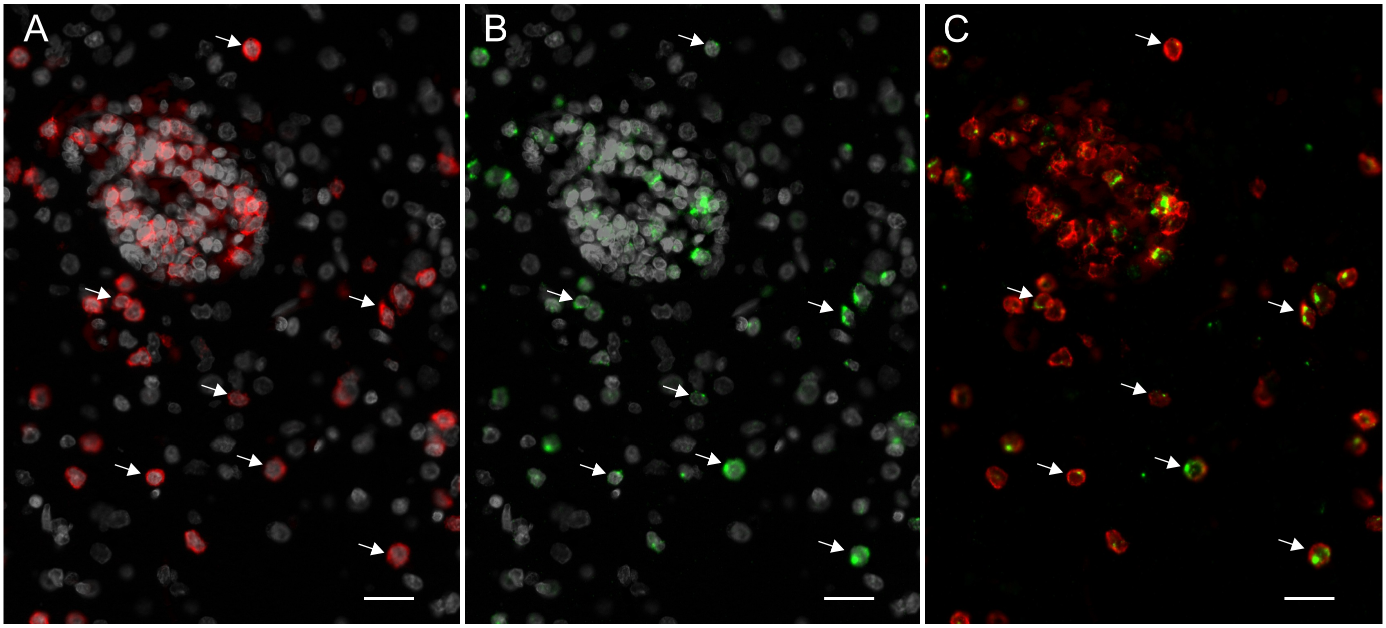

Fig. S6: LAG3 expression by CD8 T cells. (A) CD8 antibody staining of a section of resected brain tissue from Patient 738 (B) LAG3 antibody co-staining of the same section. DAPI-stained nuclei are false-colored grey. (C) Overlay of CD8 and LAG3 immunostaining. Arrows point to examples of LAG^{+} CD8 T cells.
Scale bar = 25 microns
