# Supplementary material for: Immune landscape of the affected brain in Rasmussen encephalitis
Source: Sci Rep. 2026 May 13;16:21957. doi: 10.1038/s41598-026-51295-3 (PMC13365386; doi:10.1038/s41598-026-51295-3)
Supplement: Supplementary file 8 — Supplementary Information 8. [file 41598_2026_51295_MOESM8_ESM.pdf]

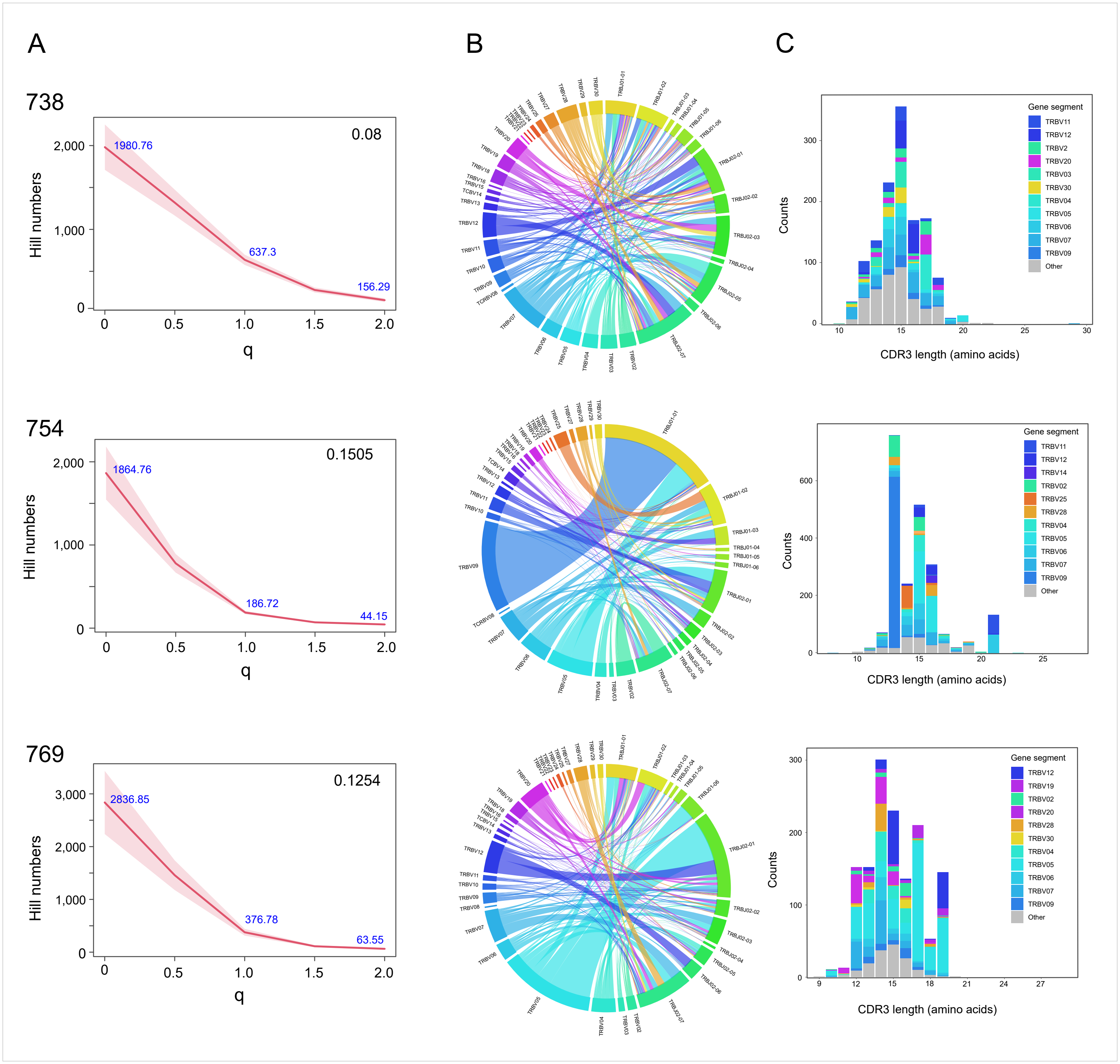

Fig. S8: (A) Hill plots showing that the diversity of T cell clonotypes in the brain surgical specimens is lowest in patient 754 and highest in patient 738 reflecting a higher clonality in patient 754 compared with patient 738 (clonality score in the top right corner of each graph). (B) Chord plots show TRBV and TRBJ gene usage is demonstrably skewed in the surgical specimens from patient 754 and patient 769. (C) Frequency histograms of Vbeta chain CDR3 lengths color coded for the proportion of frequent TRBV genes confirms the expansion of specific clonotypes (see also Table S2).
