# Supplementary material for: Immune landscape of the affected brain in Rasmussen encephalitis
Source: Sci Rep. 2026 May 13;16:21957. doi: 10.1038/s41598-026-51295-3 (PMC13365386; doi:10.1038/s41598-026-51295-3)
Supplement: Supplementary file 9 — Supplementary Information 9. [file 41598_2026_51295_MOESM9_ESM.pdf]

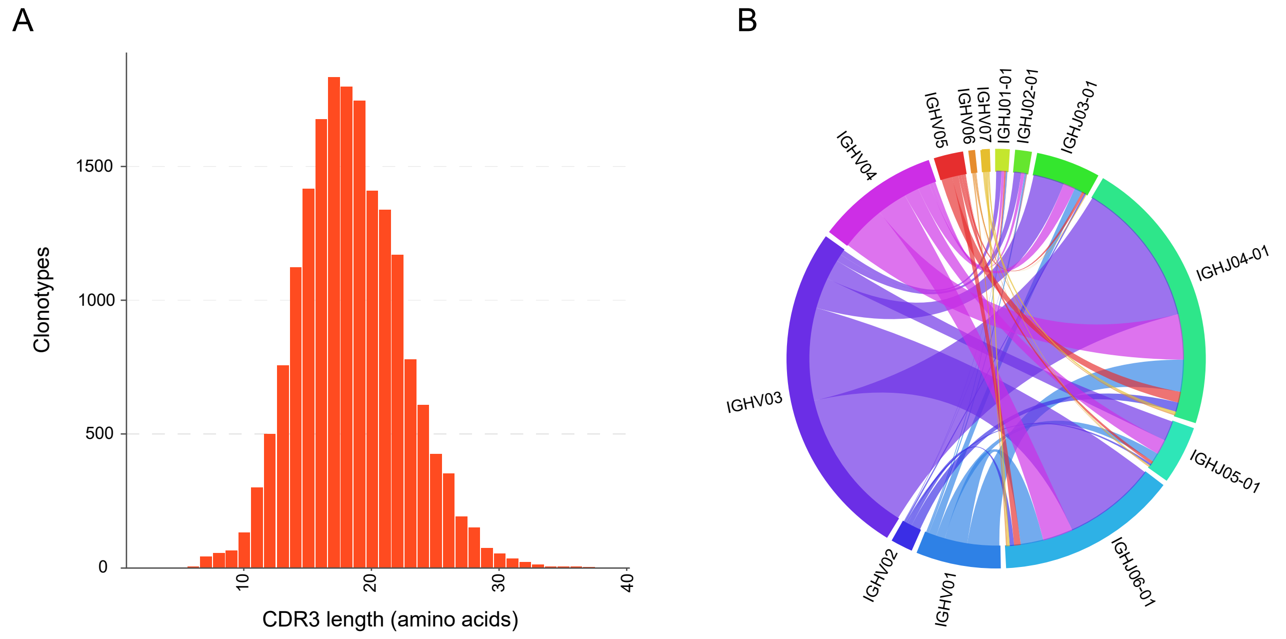

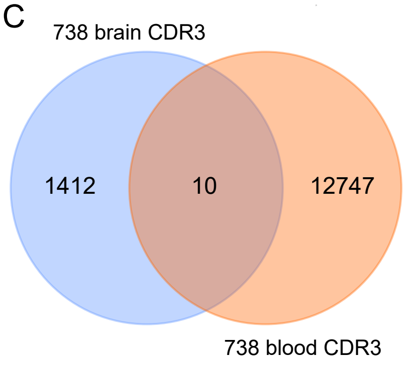

D

CDR3	Brain IGHV	Brain IGHJ	Brain Freq.(%)	Blood IGHV	Blood IGHJ	Blood Freq.(%)	p value
CAKDGSGGFGDYRRYFDYW	IGHV03-23	IGHJ04-01	0.031	IGHV03-23	IGHJ04-01	0.011	0.5304
CAKGSSTSRPYYFDYW	IGHV03-23	IGHJ04-01	0.031	IGHV03-23	IGHJ04-01	0.005	0.2862
CAKTEYYDSSGYIDYW	IGHV03-30	IGHJ04-01	0.031	IGHV03-30	IGHJ04-01	0.005	0.2862
CARALDYYGSGRLYFDYW	IGHV04-34	IGHJ04-01	0.092	IGHV04-34	IGHJ04-01	0.005	0.0079
CARDPTYDFWSGYYQPKRDGAFDIW	IGHV04	IGHJ03-01	0.031	IGHV04	IGHJ03-01	0.005	0.2862
CARDRLATNHFDGFDIW	IGHV01-18	IGHJ03-01	0.092	IGHV01-18	IGHJ03-01	0.005	0.0079
CAREIVIPPDILTGYYIDYYYYGMDVW	IGHV03	IGHJ06-01	0.031	IGHV03	IGHJ06-01	0.005	0.2862
CARGGVYCSRTSCPRGWFDPW	IGHV07-04	IGHJ05-01	0.031	IGHV07-04	IGHJ05-01	0.011	0.5304
CARGYYDSSGSPYHFYGMAVW	IGHV01-69	IGHJ06-01	0.031	IGHV01-69	IGHJ06-01	0.011	0.5304
CTRAGGYDFWIDTW	IGHV03-49	IGHJ05-01	0.031	IGHV03-49	IGHJ05-01	0.005	0.2862

Fig. S9: Immunoglobulin heavy chain (IgH) in B cells from peripheral blood of patient 738.
(A) Frequency distribution of IgH CDR3 lengths indicating that there are no over-represented clones compared with IgH chains lengths in the brain specimen (Fig. 5). (B) Distribution of IgH V and J genes. (C) Overlap of B cell clones between the samples from brain and blood. Chi-squared proportions test applied to data showed that the relative frequency of two clones was significantly higher in the brain compared with the blood.
