# Supplementary material for: Immune landscape of the affected brain in Rasmussen encephalitis
Source: Sci Rep. 2026 May 13;16:21957. doi: 10.1038/s41598-026-51295-3 (PMC13365386; doi:10.1038/s41598-026-51295-3)
Supplement: Supplementary file 10 — Supplementary Information 10. [file 41598_2026_51295_MOESM10_ESM.pdf]

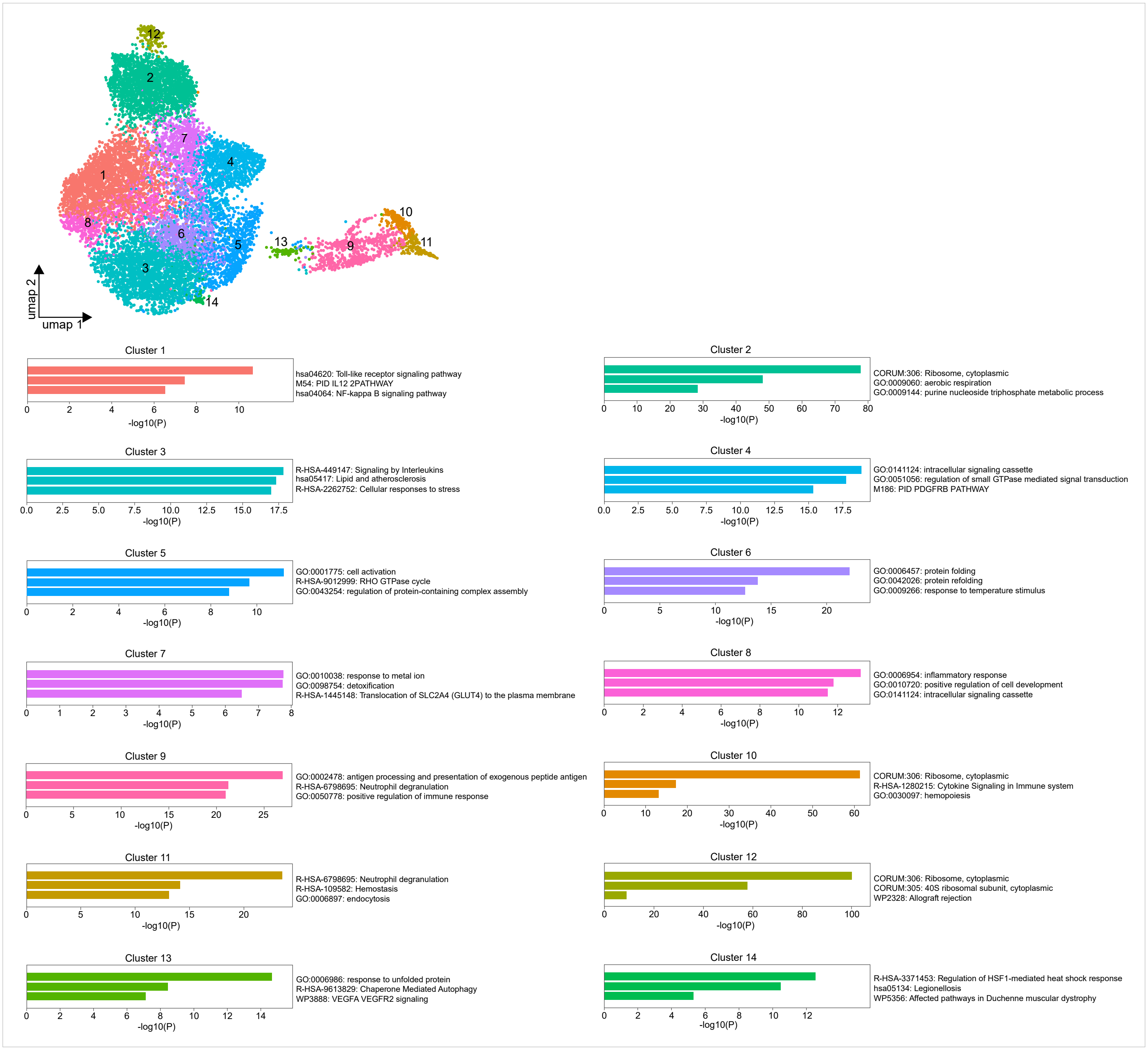

Fig. S10. Pathway analysis of myeloid clusters. Metascape was used to match the differentially expressed genes (Table S6; log2 fold-change = 0.5, ≥ 50% cells in the cluster). The top three significant pathway associations are shown.
