# Supplementary material for: Immune landscape of the affected brain in Rasmussen encephalitis
Source: Sci Rep. 2026 May 13;16:21957. doi: 10.1038/s41598-026-51295-3 (PMC13365386; doi:10.1038/s41598-026-51295-3)
Supplement: Supplementary file 11 — Supplementary Information 11. [file 41598_2026_51295_MOESM11_ESM.pdf]

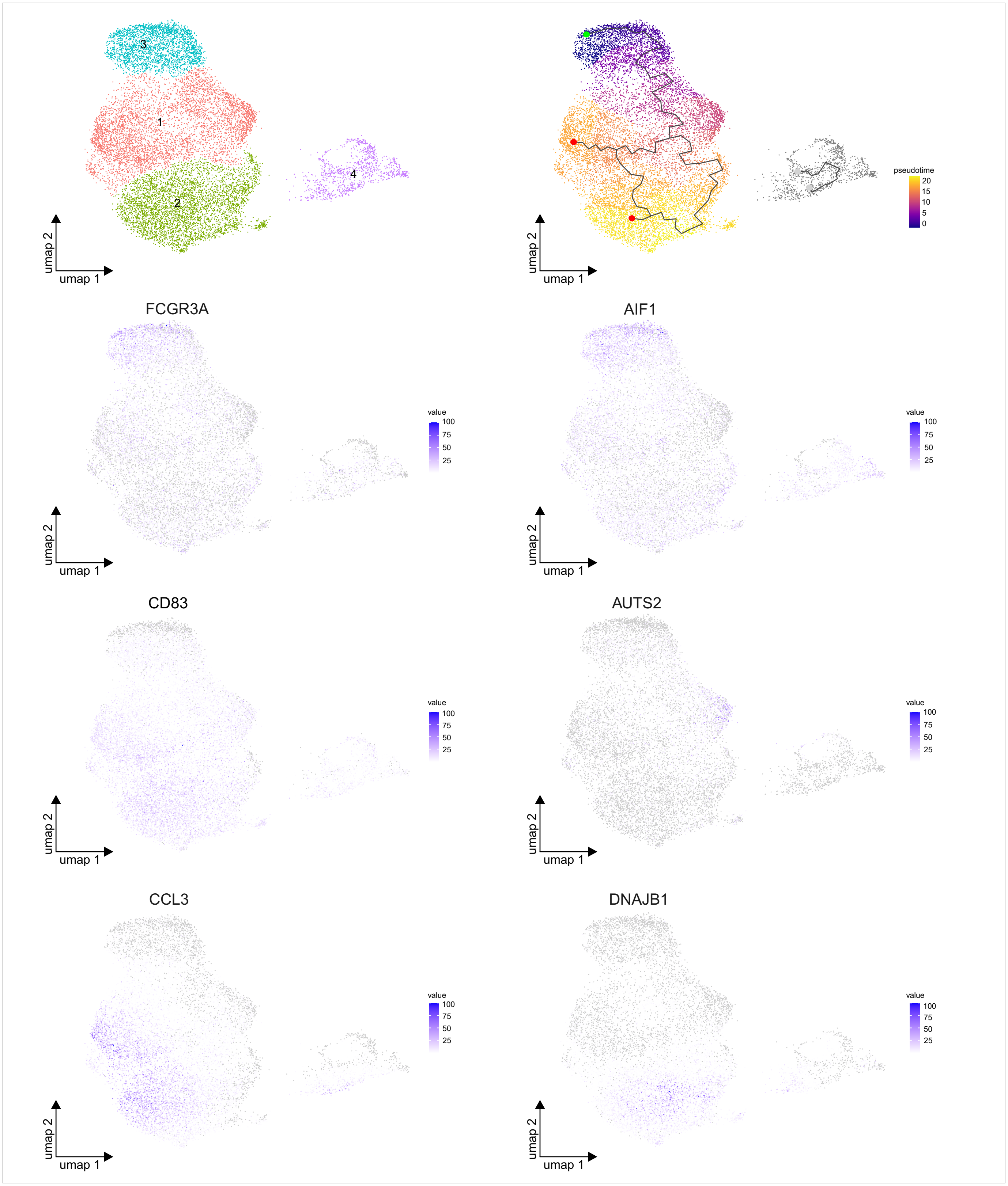

Fig. S11: Trajectory analysis using Monocle 3 shows the potential connectedness of microglia clusters. Clustering the myeloid cells with less granularity using the Leiden algorithm (resolution parameter 5e-5) resulted in 3 clusters of microglia that fit a trajectory with one start point and two end points. Feature plots show the normalized expression (expressed as a percentage) of genes that define different transitional populations (see also Fig. 6F). The autism susceptibility gene *AUTS2* identifies microglia that are not located along this calculated trajectory. In Fig. 6A cluster 4 microglia were defined in part by higher expression of AUTS2 mRNA (Table S7).
