# Supplementary material for: Immune landscape of the affected brain in Rasmussen encephalitis
Source: Sci Rep. 2026 May 13;16:21957. doi: 10.1038/s41598-026-51295-3 (PMC13365386; doi:10.1038/s41598-026-51295-3)
Supplement: Supplementary file 12 — Supplementary Information 12. [file 41598_2026_51295_MOESM12_ESM.pdf]

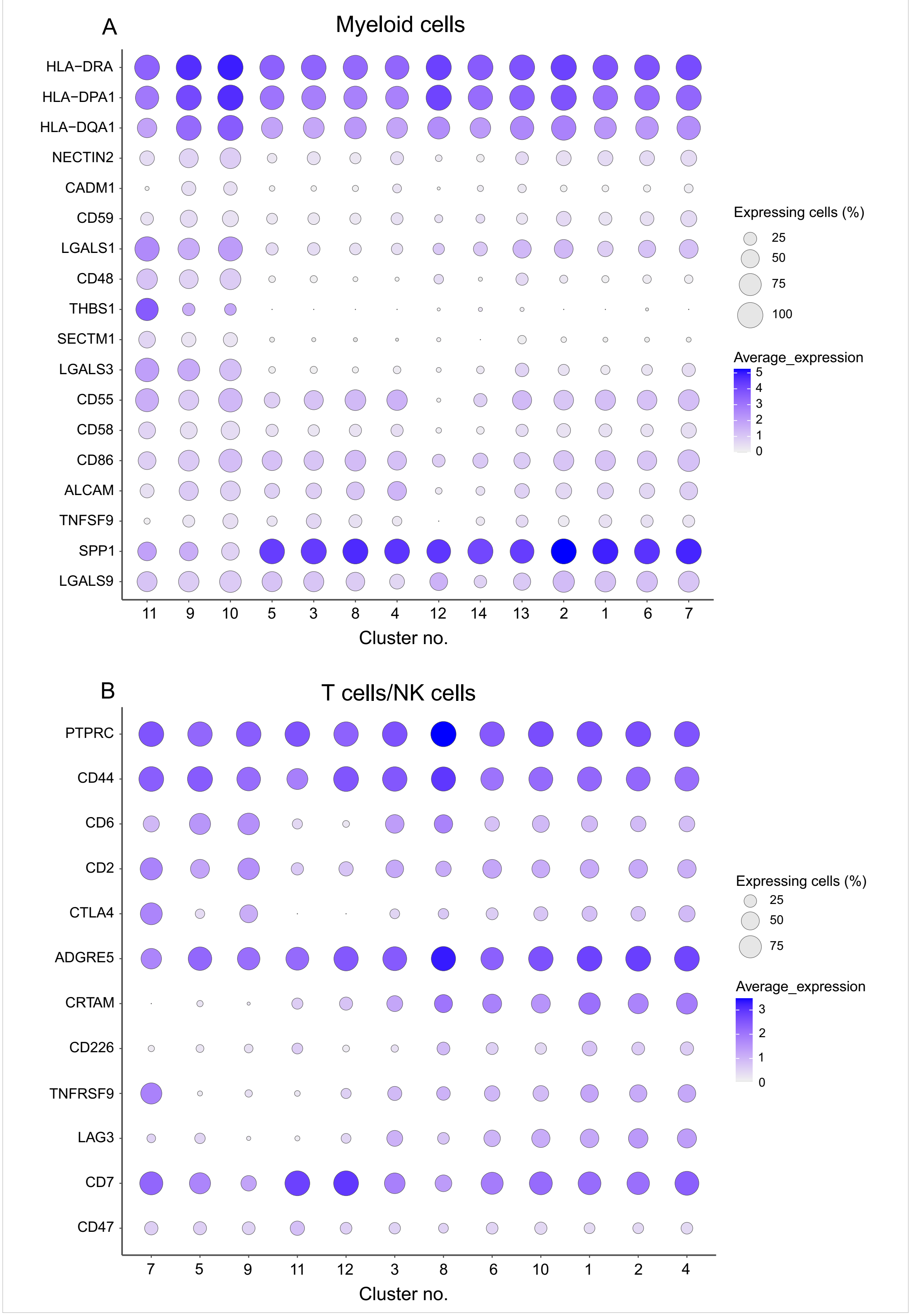

Fig. S12: (A) Bubble plot showing the normalized expression and percent expressing cells in each myeloid cluster of ligands that are predicted to bind receptors expressed by T cells and NK cells. (B) Bubble plot showing the normalized expression and percent expressing cells in each T cell and NK cell cluster of receptors that are predicted to bind the ligands expressed by myeloid cells (see Fig. 7).
