# Supplementary figures and images for: Immune landscape of the affected brain in Rasmussen encephalitis
Source: Sci Rep. 2026 May 13;16:21957. doi: 10.1038/s41598-026-51295-3 (PMC13365386; doi:10.1038/s41598-026-51295-3)

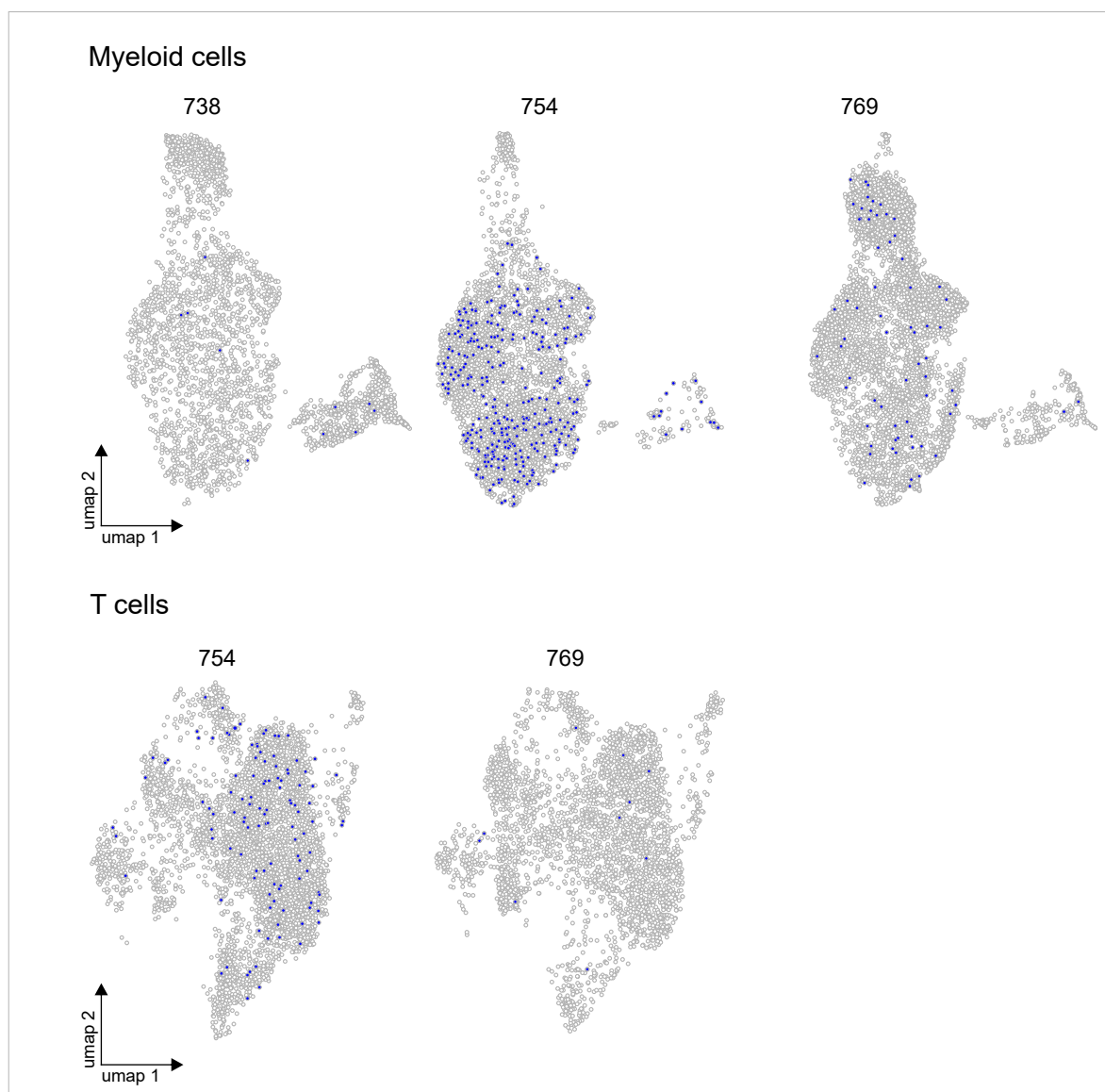

**Fig. S13:** Feature plots in which cells that express HERV-K transcripts are shown in blue.

Supplement: Supplementary file 13 — Supplementary Information 13. [file 41598_2026_51295_MOESM13_ESM.pdf]
