# Supplementary material for: Immune landscape of the affected brain in Rasmussen encephalitis
Source: Sci Rep. 2026 May 13;16:21957. doi: 10.1038/s41598-026-51295-3 (PMC13365386; doi:10.1038/s41598-026-51295-3)
Supplement: Supplementary file 16 — Supplementary Information 16. [file 41598_2026_51295_MOESM16_ESM.pdf]

Table S3: High frequency clonotypes in RE brain match rare clonotypes in the blood of individuals from two unrelated study cohorts.

ID	TRBV	TRBJ	CDR3 (nucleotide)	CDR3 (amino acid)	Frequency (%)
754	TRBV09	TRBJ01-01	TGTGCCAGCAGCGTAGATTACAGGCAGGAAGCTTTCTTT	CASSVDYRQEAFF	27
03855000013000_TCRB*	TRBV09-01	TRBJ01-01	TGTGCCAGCAGCGTGGATTACCGCCAAGAAGCTTTCTTT	CASSVDYRQEAFF	0.000187
769	TRBV05-04	TRBJ02-01	TGTGCCAGCAGCATAGGACTAGCGGGAGGAACCTACAATGAGCAGTTCTTC	CASSIGLAGGTYNEQFF	11.74
Adaptive_93†	TRBV19-01	TRBJ02-01	TGTGCCAGTAGTATTGGTCTAGCGGGGGGTACTTACAATGAGCAGTTCTTC	CASSIGLAGGTYNEQFF	0.000333
Adaptive_294	TRBV09-01	TRBJ02-01	TGTGCCAGCAGCATAGGATTGGCAGGGGGGACCTACAATGAGCAGTTCTTC	CASSIGLAGGTYNEQFF	0.000247
Adaptive_336	TRBV19-01	TRBJ02-01	TGTGCCAGTAGTATAGGACTAGCGGGAGGAACCTACAATGAGCAGTTCTTC	CASSIGLAGGTYNEQFF	0.000129
Adaptive_371	TRBV19-01	TRBJ02-01	TGTGCCAGTAGTATAGGTCTAGCGGGGGGGACCTACAATGAGCAGTTCTTC	CASSIGLAGGTYNEQFF	0.000299
Adaptive_386	TRBV19-01	TRBJ02-01	TGTGCCAGTAGTATAGGGCTAGCGGGAGGTACCTACAATGAGCAGTTCTTC	CASSIGLAGGTYNEQFF	0.000149
Adaptive_393	TRBV05-06	TRBJ02-01	TGTGCCAGCAGCATAGGACTAGCGGGAGGTACCTACAATGAGCAGTTCTTC	CASSIGLAGGTYNEQFF	0.000118
Adaptive_519	TRBV05-01	TRBJ02-01	TGCGCCAGCAGCATAGGACTAGCGGGAGGGACCTACAATGAGCAGTTCTTC	CASSIGLAGGTYNEQFF	0.000228

*https://doi.org/10.21417/RMG2022JCI
†https://clients.adaptivebiotech.com/pub/6f9fb8df-7215-47fc-9d54-7f52d973b8ee
