# Supplementary material for: Immune landscape of the affected brain in Rasmussen encephalitis
Source: Sci Rep. 2026 May 13;16:21957. doi: 10.1038/s41598-026-51295-3 (PMC13365386; doi:10.1038/s41598-026-51295-3)
Supplement: Supplementary file 17 — Supplementary Information 17. [file 41598_2026_51295_MOESM17_ESM.pdf]

Table S4: Virus-specific clonotypes.

ID	HLA-A	CDR3 amino acid	TRBV	TRBJ	Phenotype	Frequency	Trimmed CDR3	TRBV	TRBJ	HLA	organism	antigen
738	HLA-A*02	CASSLGQGPYEQYF	TRBV11-2	TRBJ2-7	CD8	0.53	ASSLGQGPYEQYF	TRBV11-2*01	TRBJ2-7*01	HLA-A*11:01	HHV4	IVTDFSVIK
	HLA-A*11	CASSIRSGETQYF	TRBV19	TRBJ2-5	CD8	0.08	ASSIRSGETQY	TRBV19*01	TRBJ2-5*01	HLA-A*02:01	influenza	GILGFVFTL
		CASSLRSSYEQYF	TRBV19	TRBJ2-7	CD8	0.08	ASSLRSSYEQY	TRBV19*01	TRBJ2-7*01	HLA-A*02:01	influenza	GILGFVFTL
		CASSRDIEAFF	TRBV7-9	TRBJ1-1	CD8	0.08	ASSRDIEAF	TRBV7-9*01	TRBJ1-1*01	HLA-A*02:01	SARS-CoV2	YLQPRTFLL
754	HLA-A*03	CASRGLNTEAFF	TRBV7-2	TRBJ1-1	Treg	0.05	ASRGLNTEAF	TRBV7-2*01	TRBJ1-1*01	HLA-A*03:01	HHV5	KLGGALQAK
	HLA-A*02	CASSVEDSSYNEQFF	TRBV9	TRBJ2-1	CD8	0.05	ASSVEDSSYNEQF	TRBV9*01	TRBJ2-1*01	HLA-A*03:01	HHV5	KLGGALQAK
		CASSVGNTIYF	TRBV9	TRBJ1-3	CD8	0.09	ASSVGNTIY	TRBV9*01	TRBJ1-3*01	HLA-A*03:01	HHV5	KLGGALQAK
769	HLA-A*03	CASSDGTGGYNEQFF	TRBV6-4	TRBJ2-1	CD8	0.071	ASSDGTGGYNEQF	TRBV6-4*01	TRBJ2-1*01	HLA-A*03:01	HHV5	KLGGALQAK
	HLA-A*31	CASSLSTDTQYF	TRBV5-4	TRBJ2-3	Treg	0.071	ASSLSTDTQY	TRBV5-4*01	TRBJ2-3*01	HLA-A*03:01	HHV5	KLGGALQAK

Table S5: Clonotypes predicted to recognize the same epitopes.

ID	CDR3	TRBV	TRBVJ	Phenotype	Frequency
738	CASSLGQGPYEQYF	TRBV11-2	TRBJ2-7	CD8	0.53
769	CASSLRQGPYEQYF	TRBV11-2	TRBJ2-7	CD8	0.07
738	CAISGSTDTQYF	TRBV10-3	TRBJ2-3	Not known	0.08
754	CASSGSTDTQYF	TRBV28	TRBJ2-3	Not known	0.05
769	CASSLSTDTQYF	TRBV5-4	TRBJ2-3	Treg	0.07
